# Decoupled carbon assimilation and growth responses to aridity in temperate deciduous oaks

**DOI:** 10.1126/sciadv.ady7139

**Published:** 2026-06-12

**Authors:** Mukund Palat Rao, Arturo Pacheco-Solana, Rong Li, Bar Oryan, Johanna E. Jensen, Milagros Rodriguez-Caton, Lily Klinek, Zoe A. Pierrat, Sophie Ruehr, Rose Oelkers, Laura E. Boeschoten, Kevin L. Griffin, M. Luke McCormack, Xi Yang, Joseph Verfaillie, Dennis Baldocchi, Jeremy Hise, Alexander J. Turner, Todd M. Scanlon, Laia Andreu-Hayles, Jan U. H. Eitel, Neil Pederson, Daniel Griffin, David Stahle, Justin T. Maxwell, Steven Voelker, Steven A. Kannenberg, Josep Peñuelas, Troy S. Magney

**Affiliations:** ^1^ Tree-Ring Laboratory, Biology and Paleo-Environment Division, Lamont-Doherty Earth Observatory of Columbia University, Palisades, NY, USA.; ^2^ CREAF, Cerdanyola del Vallès (Barcelona), Catalonia, Spain.; ^3^ Department of Plant Sciences, University of California, Davis, Davis, CA, USA.; ^4^ Cooperative Programs for the Advancement of Earth System Science, University Corporation for Atmospheric Research, Boulder, CO, USA.; ^5^ TESAF, Department of Land, Environment, Agriculture, and Forestry, University of Padova, Padova, Italy.; ^6^ Department of Environmental Sciences, University of Virginia, Charlottesville, VA, USA.; ^7^ Department of Ecology and Evolutionary Biology, Cornell University, Ithaca, NY, USA.; ^8^ Scripps Institution of Oceanography, University of California, San Diego, La Jolla, CA, USA.; ^9^ Pachama, Berkeley, CA, USA.; ^10^ Instituto Argentino de Nivología, Glaciología y Ciencias Ambientales-IANIGLA, CONICET, Mendoza, Argentina.; ^11^ NASA Jet Propulsion Laboratory, California Institute of Technology, Pasadena, CA, USA.; ^12^ Department of Geography, University of California, Santa Barbara, Santa Barbara, CA, USA.; ^13^ Biosphere Sciences & Engineering, Carnegie Institution for Science, Stanford, CA, USA.; ^14^ Department of Earth Science and Environmental Change, University of Illinois, Urbana-Champaign, Urbana, IL, USA.; ^15^ Ecology, Evolution, and Environmental Biology, Columbia University, New York, NY, USA.; ^16^ Q-ForestLab, Department of Environment, Faculty of Bioscience Engineering, Ghent University, Gent, Belgium.; ^17^ Biology and Paleo-Environment, Lamont-Doherty Earth Observatory of Columbia University, Palisades, NY, USA.; ^18^ Department of Earth and Environmental Sciences, Columbia University, New York, NY, USA.; ^19^ The Morton Arboretum, Lisle, IL, USA.; ^20^ Biology Department University of Illinois Chicago, Chicago, IL, USA.; ^21^ Department of Environmental Science, Policy and Management, University of California, Berkeley, Berkeley, CA, USA.; ^22^ Hise Scientific, Somers, NY, USA.; ^23^ Department of Atmospheric and Climate Science, University of Washington, Seattle, WA, USA.; ^24^ Catalan Institution for Research and Advanced Studies (ICREA), Barcelona, Spain.; ^25^ Department of Natural Resources and Society, University of Idaho, Moscow, ID, USA.; ^26^ Harvard Forest, Harvard University, Petersham, MA, USA.; ^27^ Geography, Environment, & Society, University of Minnesota, Minneapolis, MN, USA.; ^28^ Saint Anthony Falls Laboratory, University of Minnesota, Minneapolis, MN, USA.; ^29^ Department of Geosciences, University of Arkansas, Fayetteville, AR, USA.; ^30^ Department of Geography, Indiana University, Bloomington, IN, USA.; ^31^ College of Forest Resources and Environmental Science, Michigan Technological University, Houghton, MI, USA.; ^32^ Department of Biology, West Virginia University, Morgantown, WV, USA.; ^33^ CSIC, Global Ecology Unit CREAF-CSIC-UAB, Bellaterra, Catalonia, Spain.; ^34^ Department of Forest Management, University of Montana, Missoula, MT, USA.

## Abstract

The magnitude of the terrestrial carbon sink remains a key uncertainty in future climate projections, in part due to poorly understood links between carbon uptake and its allocation to woody biomass in vegetation. Here, in this study, we show that photosynthesis and aboveground growth occur asynchronously across diel to seasonal scales in eight North American oak species. Across 137 tree ring sites, current-year annual growth was insensitive to climate variability after midsummer despite 26 to 36% of annual gross primary productivity (GPP) occurring during this period. Hourly GPP flux and growth measurements at four sites spanning seven site years further demonstrate that wood formation ceases earlier than photosynthesis and is restricted to periods of low atmospheric aridity and temperature. This photosynthesis-growth decoupling intensifies with interannual variability in vapor pressure deficit (*r* = 0.86, *P* < 0.05), suggesting that by assuming tight coupling between photosynthesis and woody biomass, current earth system models may overestimate long-term carbon sequestration in forests.

## Teaser

Long-term carbon sequestration as woody biomass in oaks is decoupled from photosynthesis and more sensitive to climate change.

## INTRODUCTION

The terrestrial carbon cycle is one the least constrained components of the global carbon cycle, resulting in uncertain projections of future carbon dioxide (CO_2_) and global climate (*1*–*3*). Terrestrial ecosystems including forests are estimated to offset nearly a third of annual CO_2_ emissions from human fossil fuel burning and land-use change (*3*). Forests act as carbon sinks by assimilating carbon through photosynthesis, referred to as gross primary productivity (GPP) at ecosystem scales, and allocating a fraction of this carbon to long-term storage in woody biomass and soils (*4*, *5*). Earth system models (ESMs) generally project a stable to increasing terrestrial carbon sink through this century as a result of higher atmospheric CO_2_ leading to enhanced photosynthesis in vegetation (CO_2_ fertilization) and higher carbon storage in woody biomass and soils (*6*, *7*). Many ESMs, however, rely on a photosynthesis centric approach to forest carbon cycling postulating that the carbon sink capacity of forests is predominantly limited by their ability to assimilate carbon (*8*–*11*).

The connection between carbon assimilation and allocation is complex. Following its assimilation, carbon is distributed among various plant processes and pools including respiration, foliage, reproduction, nonstructural carbohydrates (NSCs), root mycorrhizae and exudates, volatile compounds, defensive compounds, and above and belowground woody biomass growth (*12*–*19*). As the fractional allocation of carbon across these sinks is highly dynamic, carbon assimilation may not necessarily be a good proxy for its allocation toward woody biomass growth and the “photosynthetic season” may not necessarily be the same as the woody biomass “growing season” (*20*–*22*). Since woody tissues represent one of the longest-lived carbon pools in terrestrial ecosystems, resolving the degree of photosynthesis-growth coupling is essential for accurate prediction of future carbon-climate feedbacks (*6*, *22*–*25*).

Photosynthesis can be reasonably well estimated using in situ measurements, remote sensing, and eddy covariance monitoring of CO_2_ fluxes above the canopy (*26*–*28*). In contrast, quantifying woody biomass growth—an inherently microscopic process occurring within the cambial zone—is far more challenging across scales, from individual trees to ecosystems (*10*, *29*). While empirical relationships between environmental variables and photosynthesis are well established, the same cannot be said for woody biomass growth (*10*, *30*). Flux tower–based eddy covariance, tree ring, and forest inventory data suggest that carbon assimilation and allocation to aboveground woody biomass growth (also referred to as secondary radial growth) are equivocally correlated at interannual timescales (*31*–*37*). The mechanisms underlying the lack of consistent photosynthesis-growth or source-sink coupling across environmental and ecological gradients, however, remain unresolved (*22*, *34*, *38*).

**Fig. 1.**
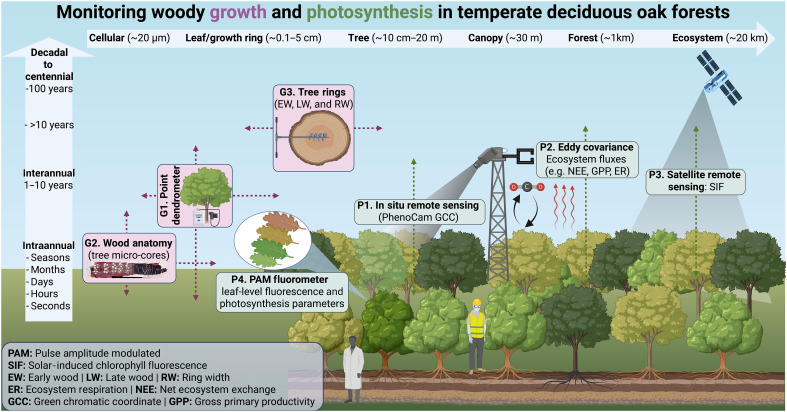
Experimental design used to evaluate coupling between photosynthesis (P) and aboveground woody biomass growth (G) across multiple North American temperate deciduous oak forest sites. Measurements are categorized across a gradient of spatiotemporal scales from the cellular to ecosystem levels and from seconds to centuries. The map in fig. S1 shows locations of four high-resolution monitoring sites, study years, species, and measurements made at each site (i. Morton Arboretum–IL, ii. Lamont Sanctuary–NY, iii. Pace Forest–VA, and iv. Tonzi Ranch–CA). Photosynthesis-related data are collected using in situ remote sensing from PhenoCams (P1) at all four sites, eddy covariance (P2) at Pace-VA and Tonzi-CA, satellite remote sensing (P3) at Morton-IL and Lamont-NY, and leaf-level chlorophyll fluorescence (P4) at Lamont-NY only. Growth data are collected using point dendrometers (G1) at all four sites and wood anatomy (G2) at Lamont-NY only. Figure 2 presents locations and analyses based on 137 tree-ring sites (G3). We study eight oak species QUAL: *Quercus alba*, QUBI: *Quercus bicolor*, QUMO: *Quercus montana*, QURU: *Quercus rubra*, QUCO: *Quercus coccinea*, QUPA: *Quercus palustris*, QUFA: *Quercus falcata*, and QUDG: *Quercus douglasii* in total. Created in BioRender. Rao, M. (2026) https://BioRender.com/p0yedtx.

Although many ESMs assume vegetation to be photosynthesis or carbon source limited, carbon allocation to sink processes such as plant growth can be more sensitive to water availability than photosynthesis. Diminished turgor, driven by reductions in hydraulic conductance to expanding cambial cells under drought can rapidly inhibit growth activity independent of carbon availability, while photosynthetic rate declines driven by stomatal closure and enzyme kinetics occur more gradually (*39*–*45*). As temperature, drought, and atmospheric vapor pressure deficit (VPD) continue to rise under climate change (*46*–*49*), divergent responses of photosynthesis and growth may lead to carbon allocation shifts away from tree woody biomass to more transitory carbon pools that have lower residence times (*44*, *50*–*52*). Understanding the fate of assimilated carbon thus requires a mechanistic view of source-sink dynamics that accounts for both photosynthesis and growth responses. Colocated hourly to daily resolution photosynthesis and growth data spanning individual trees to entire ecosystems can provide a mechanistic understanding of source-sink coupling at the spatiotemporal scales at which these processes occur. However, these estimates are extremely rare (*9*, *10*, *53*, *54*). To better understand and predict the carbon sink capacity of forests in a future climate system, we examine the degree of temporal coupling between photosynthetic carbon assimilation and its allocation to aboveground woody biomass growth in North American temperate deciduous oak (*Quercus* spp.) forests. We do so across intra to interannual temporal scales and from cellular to satellite remote sensing spatial resolutions (see Fig. 1, fig. S1, and Materials and Methods).

Oaks are an ecologically and economically important clade, occasionally referred to as the “most important woody genus in the Northern Hemisphere,’ and its members are foundation species across many North American forest regions (*55*–*57*). Specifically, we ask the following: (i) How distinct are the photosynthetic season and aboveground woody biomass growing season for oaks? (ii) Do atmospheric aridity and air temperature more strongly constrain growth than photosynthesis across diel to seasonal timescales? We hypothesize that in oaks, (i) photosynthesis and growth are often temporally decoupled, (ii) high VPD and temperature limits growth more strongly than carbon assimilation, and (iii) intraannual variation in aridity (VPD or temperature-precipitation interaction) corresponds to the degree of seasonal decoupling. We define growth as cellular division and expansion in tree cambia that results in new vascular tissue and increases in radial stem thickness and aboveground woody biomass. Our results provide insights into photosynthesis-growth asynchrony in oak forests and guidance for improving carbon allocation dynamics in ESMs under future climate scenarios.

## RESULTS

### Aboveground annual radial growth is insensitive to late summer and autumn climate despite sustained carbon assimilation

**Fig. 2.**
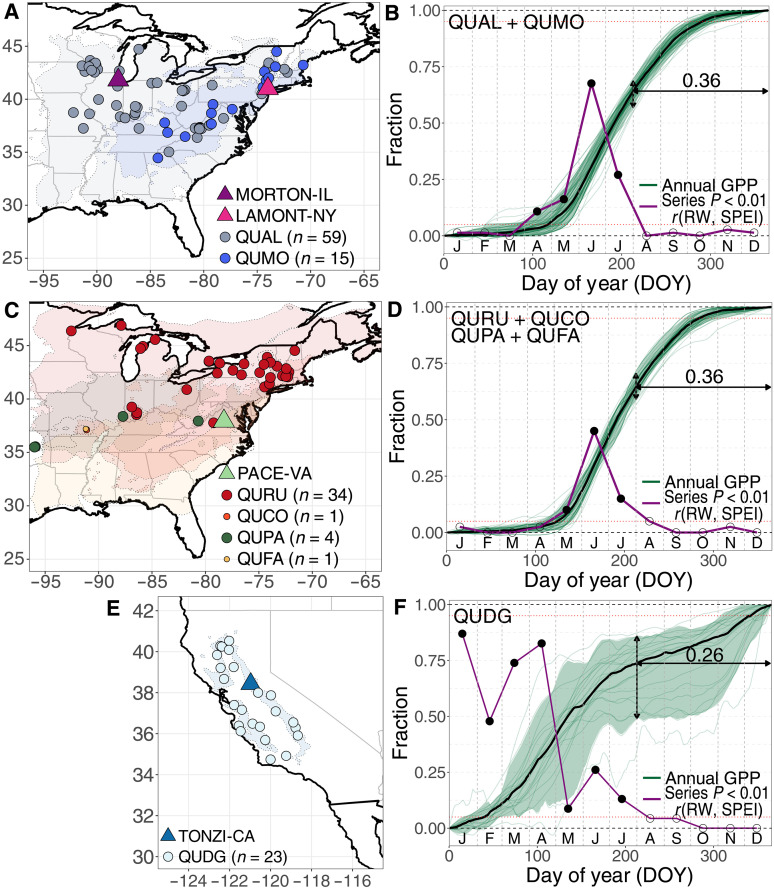
Inferring temporal decoupling between photosynthesis and growth by comparing the annual fraction of cumulative GPP with the climate sensitivity of annual tree RW for 137 North American oak (*Quercus* spp.) chronologies and seven species. (**A**) Locations of 74 tree-ring series of the white oak group *Quercus* sect. *Quercus*, represented by QUAL (*n* = 59) and QUMO (*n* = 15) and their distribution ranges (in gray and blue, respectively). (**B**) Phenology of the fraction of annual GPP in 2021 as a function of the Day of year (DOY) across all tree-ring sites and species in part (A). Bold black line and green shading represent the median, 5th, and 95th percentiles of cumulative GPP, and light green lines describe GPP at each site. The number (0.36) indicates the median fraction of annual GPP (i.e., 36% annual GPP) assimilated after July (i.e., August to December). Dotted red horizontal lines indicate GPP fractions of 0.05 (start of photosynthetic season) and 0.95 (end of photosynthetic season). Purple solid line describes the fraction of tree-ring chronologies of 74 total that have a significant correlation (*P* < 0.01, two-sided Student’s *t* test) between annual RW and 1-month SPEI for the same year between 1950 and the last year of each tree-ring series (also see figs. S2 and S3). Filled circles in black represent months where a minimum of 5% of all 74 series showed a significant correlation (*P* < 0.01, two-sided Student’s t test) between SPEI and annual radial growth. Subplots (**C**) and (**D**) are similar to (A) and (B) but for 40 tree-ring chronologies of the red oak group, *Quercus* sect. *Lobatae*, represented by four species QURU, QUCO, QUPA, and QUFA with 0.36 as median post-July annual GPP median fraction and (**E**) and (**F**) are for 23 QUDG tree-ring chronologies in California with 0.26 as median post-July annual GPP fraction. Triangles on maps show locations of high-resolution monitoring sites and described in fig. S1 along with species codes. Tree-ring metadata is provided in table S1.

To assess the timing of photosynthesis and growth in temperate deciduous oak forests, we estimated daily GPP for 137 tree-ring sites using satellite-derived solar-induced chlorophyll fluorescence calibrated against half-hourly flux tower data and downscaled to 500-m resolution (*58*). These data show that the photosynthetic season spanned late April to October (~7 months) in the Eastern US and mid-February to mid-December (~10 months) in California (Fig. 2). Across all sites, the late summer through autumn season contributed more than a quarter of annual GPP. Approximately 36% (30 to 43%) of the annual GPP in the Eastern US sites and 26% (14 to 51%) of the annual GPP at the California sites occurred after July (i.e., between August and December).

Despite this sustained carbon uptake, climate conditions during this late-season period had little to no influence on annual aboveground radial growth. At 114 Eastern US tree-ring series (that included six species), the highest and most consistent positive correlations between total annual tree-ring width (RW) and 1-month Standardized Precipitation Evapotranspiration Index (SPEI) were from spring to midsummer between May, June, and July for the current year (*t* + 0) (Fig. 2 and figs. S2 and S3). In contrast, the highest and most consistent RW-SPEI correlations at the 23 California sites were observed between winter and spring for a period from December of the previous year (*t* − 1) through current year (*t* + 0) April, with weaker correlations extending into midsummer (June to July). In other words, wet and cool spring to midsummer in the Eastern US and wet and cool winter to spring in California favor enhanced tree growth in the current year across sites and oak study species. Across all sites, post-July climate showed negligible impact on growth: Fewer than 5% of sites exhibited significant RW-SPEI correlations (i.e., *P* < 0.01) after current year July.

Together, these findings reveal a substantial disconnect: Twenty-six to 36% of annual photosynthesis occurs after July (i.e., between August and December), yet late-season climate plays little to no role in determining annual woody growth in the current year. While there is a distinction between when a tree grows and the climate window to which annual growth is most sensitive, these results suggest the phenology of aboveground radial growth and carbon assimilation are likely decoupled from each other. These patterns motivate mechanistic investigation of source-sink decoupling at a higher temporal resolution.

### Despite a synchronous start, the growing season ends before the photosynthetic season

**Fig. 3.**
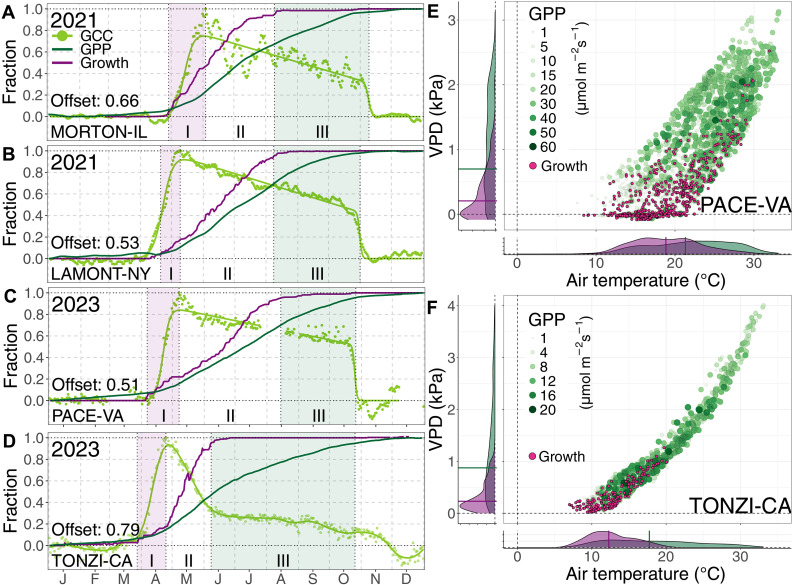
Seasonal photosynthesis-growth decoupling at four oak forest sites in North America. Growth occurs over a narrower climatic niche of cooler temperature and lower atmospheric aridity (VPD) than does GPP. (**A**) Phenology of PhenoCam-derived canopy GCC (light green), cumulative fraction of annual GPP (dark green), and point dendrometer–derived cumulative fraction aboveground radial growth (in purple) at Morton Arboretum–IL in 2021. Active season is divided into three phases (Table 1). Phase I and phase III are highlighted in purple and green, respectively. (**B**) to (**D**) are similar to (A) but for Lamont Sanctuary–NY in 2021, Pace Forest–VA in 2023, and Tonzi Ranch–CA in 2023. *X* axis labels represent abbreviated months of the year. Offset is a metric of the seasonal decoupling between GPP and growth (see fig. S5). (**E**) Environmental sensitivity of GPP (green) and growth (purple) as a function of air temperature and VPD at Pace Forest–VA. Circle sizes and color scale with GPP. Kernel density plots show temperature and VPD distributions for growth and GPP along with vertical bars for medians. (**F**) As in (E) but at Tonzi Ranch–CA. Comparisons in (E) and (F) are for all hours during seasonal phase II (7 May 07 to 14 Aug. at Pace Forest–VA; 25 Apr. to 08 June at Tonzi Ranch–CA in 2023). Growth occurs when a dendrometer records a new maximum value that supersedes all prior maxima [Zero-Growth Concept, Zweifel *et al.* (*59*)]. Growth occurrences are shown for all dendrometer-monitored trees at each site in (E) and (F) and median across all trees in (A) to (D). Note that axis scales differ in subplots (E) and (F).

Across all four high-resolution monitoring sites (fig. S1), foliar expansion of the canopy, GPP, and growth commenced together (Fig. 3 and figs. S4 to S7). The start of the active season, the first date when either cumulative annual growth or GPP exceeded 5%, occurred in the spring between late March and late April at all the four sites. Despite this concurrent start of canopy expansion, photosynthesis, and growth, the fraction of annual growth consistently surpassed the fraction of annual GPP (Fig. 3). Moreover, growth activity consistently ended 2 to 4 months before photosynthetic activity (Fig. 3).

Canopy development (foliar expansion) was deduced using phenological camera (PhenoCam) green chromatic coordinate (GCC), and GPP was derived using flux tower–based eddy covariance at Pace Forest–VA and Tonzi Ranch–CA and satellite remote sensing at Morton Arboretum–IL and Lamont Sanctuary–NY (see Materials and Methods). We partitioned point dendrometer changes into irreversible radial woody biomass growth due to the building and expansion of new cells and excluded reversible stem shrinkage induced by tree water deficit (TWD) using the zero growth concept (*59*). At the seasonal scale, we divided the active season into three main phases described in further detail in Table 1: phase I, leaf expansion (primary growth) together with aboveground radial growth (secondary growth) but GCC inferior to its annual maximum; phase II, concurrent GPP and radial growth with maintenance of fully expanded foliage and canopy; and phase III, continued canopy greenness and GPP but down-regulated radial growth, followed by canopy senescence. Across the four sites, the total fractional period of offset, which represents the fraction of the active season where growth and GPP were decoupled (i.e., phases I and III, excluding phase II), ranged from ~50% at Pace Forest–VA to 80% at Tonzi Ranch–CA (fig. S5). As the offset metric indicates the fractional length of season when GPP-growth phenologies are decoupled, an 80% offset therefore means that growth and GPP are decoupled for 80% of the time during the active season. In other words, the fractional length of phase II when GPP and growth co-occur synchronously represents 20% of the active season. We include phase I in this calculation as GPP rates are relatively low during the period before full leaf expansion, and the fastest GPP rates only commence in phase II (Fig. 3 and fig. S4). This pronounced decoupling, captured at hourly-to-daily resolution, aligns with patterns inferred from annual tree-ring records and underscores a systematic temporal disconnect between seasonal carbon uptake and its allocation to woody biomass. In the next section, we explore the climatic drivers of this decoupling across diel to seasonal timescales.

**Table 1.** Three phases of photosynthesis-growth coupling across seasonal scales observed at high-resolution monitoring sites. Seasonal scale results describe Fig. 3 and figs. S4 to S12. Leaf level photosynthetic capacities and woody anatomical insights are described in further detail in texts S1 and S2, respectively.

Phase	Timing	Physiology
**I**	Spring	Rapid aboveground radial woody biomass growth together with canopy expansion, gradually increasing GPP (Fig. 3 and figs. S4 to S8). Leaves develop their photosynthetic capacities during this phase (text S1 and figs. S9 and S10). This is further supported by microcore images and quantitative woody anatomy data based on xylogenesis (text S2 and figs. S11 and S12). Trees are primarily forming earlywood during this phase in the spring. The formation of large vessels in the earlywood leads to a steep seasonal increase in conductive area during phase I, measured by the relative conductive area over time (RCTA). Across sites (see fig. S5): Fractional length of phase I (PI) relative to active season is between 10 and 20%; Percent of annual growth in phase I (PI-Gr) is between 18 and 49%.
**II**	Early summer	Co-occurring aboveground radial woody biomass growth and GPP and maintenance of green foliar canopy based on GCC (Fig. 3 and figs. S4 to S8). Photosynthetic capacity is also maintained (text S1 and figs. S9 and S10). Trees are forming latewood with smaller vessels and lower conductive area. RCTA values plateau during this phase, indicating a transition from rapid earlywood vessel expansion to the formation of narrower latewood vessels, which is consistent with the anatomical observations of reduced vessel size and ring maturation across sampling dates (text S2 and figs. S11 and S12). At the end of phase II, annual growth for the year is complete (reaches 95% of the annual maximum). Annual cumulative GPP lags behind growth and reaches 70 to 76% at the three Eastern US sites and ~52% at the Southwestern US site at Tonzi Ranch–CA.
**III**	Late summer to autumn	Annual growth is complete (dendrometers reached 95% of their annual maximum). However, GPP continues and foliar canopies (GCC) and its photosynthetic capacities are maintained up to the time when leaf senescence begins (Fig. 3 and figs. S4 to S10). Phase III is shorter at the three Eastern US sites than the Southwestern US sites in California (Fig. 3 and figs. S4 and S5). Microcores do not appear to show active cell formation, division, or lignification in the cambia (text S1 and figs. S11 and 19). The longer phase III at Tonzi Ranch–CA is likely attributable to its winter-dominated precipitation regime and the high degree of plant water stress during the summer that reduces GPP (*89*). Across sites (see fig. S5): Fractional length of phase III relative to active season is between 36 and 66%; Percentage GPP assimilated in phase III is between 24 and 48%.

### Aridity as a driver of photosynthesis and growth decoupling mediated by tree water status

At two eddy covariance flux tower sites equipped with dendrometers, Pace Forest–VA and Tonzi Ranch–CA, woody growth occurred under significantly lower VPD and temperature than photosynthetic carbon assimilation (Fig. 3, E and F, *P* < 0.01, 1000 bootstrapped samples of median with replacement). At Pace Forest–VA in 2023, the median temperature and VPD were 21.3°C and 0.70 kPa for GPP, respectively, and 18.9°C and 0.20 kPa for growth. A similar separation in the VPD-temperature environmental niche was also evident at Tonzi Ranch–CA. The median temperature and VPD at which GPP occurred at Tonzi Ranch–CA were 17.8°C and 0.88 kPa, respectively, considerably higher than the median temperature and VPD at which growth occurred, which were 12.3°C and 0.23 kPa, respectively, in 2023. These patterns were most evident during the middle of the season (phase II), when leaves were fully expanded and photosynthesis and growth co-occurred most strongly. However, similar results were found for GPP-growth comparisons that included phase I, where growth occurs before full leaf expansion and GPP remains below its seasonal maxima (fig. S6). We excluded phase III here, in Fig. 3 (E and F) and fig. S6, to limit comparisons between the environmental niches of GPP and growth to only include the period when both processes occurs as little to no growth happens during phase III despite continued GPP.

**Fig. 4.**
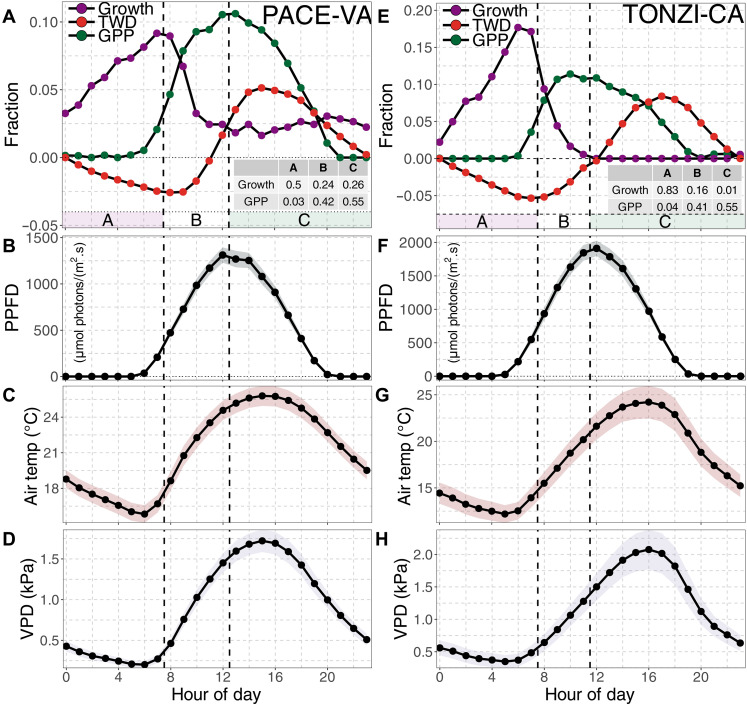
Photosynthesis-growth decoupling at the diel scale. Growth occurs between late night and early morning (00:00 to 07:00 hours) over a narrower climatic niche of cooler temperature and lower VPD than GPP, which peaks at or just before solar noon. (**A**) Fraction of growth occurrences (green dots), fraction of GPP, standardized TWD, (**B**) photosynthetic photon flux density (PPFD, μmol photons·m^−2^·s^−1^), (**C**) air temperature (°C), and (**D**) VPD (kPD) across hours of the day (00:00 to 23:00 hours) during seasonal phase II (7 May to 14 Aug) at Pace Forest–VA in 2023. Shading in subplots (B), (C), and (D) ± 2 SEM. Subplots (**E**) to (**H**) follow the same order but are for seasonal phase II (25 Apr to 8 June) at Tonzi Ranch in 2023. See fig. S13 for diel photosynthesis-growth decoupling across phase I and phase II seasonal scales. Growth occurrence fraction is calculated across hours when a growth occurrence is registered on all monitored trees following the zero growth concept [Zweifel *et al.* (*59*)]. GPP and growth fractions sum to one. Mean daily standardized TWD curve is derived by scaling daily TWD by the daily maximum TWD in comparison to midnight TWD. Mean daily standardized TWD is therefore related to reversible stem radial change. Negative and positive standardized TWD values indicate an expanding and contracting stem, respectively, relative to TWD at midnight. We divide photosynthesis-growth coupling at the diel scale into three phases (Table 2). The table in subplots (A) and (E) describe the fraction of seasonal growth and GPP during diel phases A, B, and C. Note different *y* axis scales between left and right panels.

The difference in the environmental sensitivity of growth and GPP to temperature and VPD at the seasonal scale (Fig. 3, E and F) is likely attributable to differences in the timing and environmental sensitivity of growth and GPP activity at the diel scale (Fig. 4 and fig. S13). Analogous to coupling at the seasonal scale, we categorized photosynthesis-growth coupling at the diel scale into three phases (A, B, and C) (Table 2). At both Pace Forest–VA and Tonzi Ranch–CA, we found that growth activity at the diel scale is most likely to occur during phase A between late night to early morning (00:00 to 07:00 hours) when daily standarized TWD, VPD, and temperature were the lowest. During this period, trees rehydrate stem tissues depleted during daytime transpiration, consistent with TWD decline and xylem, cambial, and phloem refilling (*59*–*61*). In phase B, GPP commences synchronously with increasing light availability and peaks by solar noon. However, during this same window, growth activity declines sharply as increased VPD and temperatures enhance TWD and likely lead to decreased turgor in the cambial cells. By solar noon, growth likelihood reaches a daily minimum, suggesting that increasing hydraulic stress strongly inhibits cell expansion (*59*). The likelihood of growth continues to remain low during the remainder of the day during phase C, as stems begin to rehydrate again (decreasing TWD) into the evening after 16:00 hours local time. At Pace Forest–VA, a relatively mesic site (fig. S8), 50% of all growth activity occurs during early morning (phase A) at the diel scale with the remaining growth activity being split evenly between phase B and phase C. The distribution of growth activity at Tonzi Ranch–CA, a semiarid site with little precipitation during the active season, is more strongly skewed toward phase A with 83% of growth activity occurring before 7:00 hours. GPP activity is evenly distributed between phase B and phase C at both sites (~41 to 55%), although phase B between early morning and peak GPP activity around solar noon is shorter than phase C.

**Table 2.** Three phases of photosynthesis-growth coupling across diel scales observed at high-resolution monitoring sites. Diel scale results are presented in Fig. 4 and fig. S13.

Phase	Timing	Physiology
**A**	Early morning (before 7 hours)	Rapid growth, low GPP due to the lack of available light, and TWD is low as stems rehydrate. VPD and temperature are at their daily minima. Majority of growth activity takes place in this diel phase (50% at Pace and >80% at Tonzi).
**B**	Morning to solar noon	Co-occurring growth and photosynthesis. GPP increases with light availability, but increasing TWD or plant water stress begins to inhibit growth. GPP activity is strongest during this phase, and close to half of seasonal GPP occurs between 7 hours and solar noon (peak photosynthetic photon flux density, PPFD) at the diel scale.
**C**	Post-solar noon to night	GPP activity continues but its strength decreases, and growth remains down-regulated likely due to persistently high TWD.

Soil moisture availability (water supply) in addition to VPD (atmospheric water demand), precipitation, and temperature can be an important driver of GPP and growth and also modulate their respective phenologies (*62*). To evaluate the potential role of soil water content (SWC) in specifically influencing photosynthesis-growth decoupling, we contrasted the climatic niches of GPP and growth relative to SWC at Tonzi Ranch–CA as it was the only site with all the necessary data available (fig. S14). Unlike the results obtained for temperature and VPD (Fig. 3, E and F, and fig. S14), kernel density distributions for growth and GPP as a function of SWC across three different depths with replicates indicated broad overlap. In other words, the SWC climate niche is similar for GPP and growth. Although future work is needed to test this across more sites and climates, this suggests that while soil moisture may contribute to setting the boundary conditions for both GPP and growth, it may not drive the decoupling between them in the same way VPD or temperature does.

We also found strong and significant positive relationships between daily tree water status, based on the normalized maximum daily shrinkage (MDS_norm_) metric (*62*) and both daily maximum VPD and air temperature at Tonzi Ranch–CA during the active season from seasonal phase I through III (*P* < 0.01 two-sided *t* test; figs. S15 and S16). This indicates that across diel to seasonal scales, a combination of warm temperatures and high VPD likely inhibit growth activity due to increased water stress and decreased turgor in tree cambia (*62*). Further, it is likely that such inhibition of growth occurs faster than reductions in GPP to environmental stress (Fig. 3, E and F) (*41*) .

**Fig. 5.**
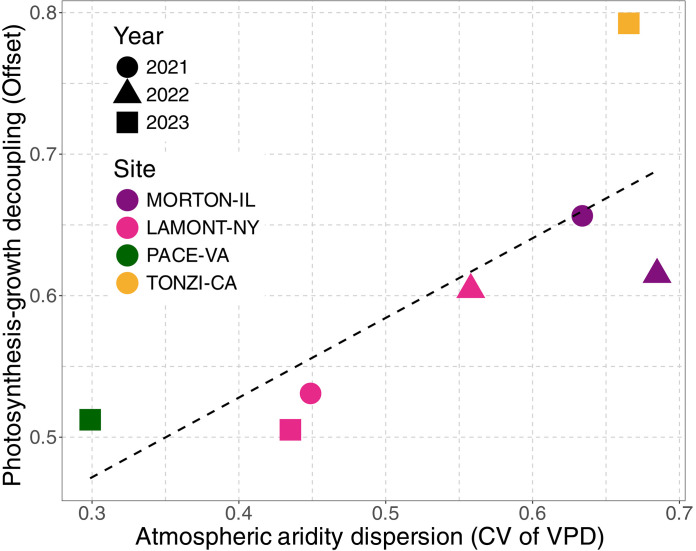
Annual dispersion in atmospheric aridity increases photosynthesis-growth decoupling. Relationship between the annual dispersion atmospheric aridity, measured by the annual CV of mean monthly VPD and the fractional period of photosynthesis-growth offset (Offset). Offset represents the fraction of the active season when growth and GPP are decoupled or not fully co-occurring (fractional length of seasonal phase I and phase III, relative to phases I, II, and III). Photosynthesis-growth decoupling increases as annual dispersion of VPD increases (Spearman *r* = 0.86, *P* < 0.05). This suggests that as aridity becomes more variable interannually, the seasonal time period of the active season that is favorable for both growth and photosynthesis to co-occur decreases (also see figs. S17 and S18). Note that in the legend, colors correspond to sites and shapes to site-years.

As growth consistently occurred at cooler and more humid conditions than GPP, we evaluated whether the dispersion in intraannual variability of environmental factors (VPD, temperature, and precipitation) could explain the degree of seasonal decoupling between growth and photosynthesis (Offset parameter) across all sites. We found a significant positive relationship between Offset and the coefficient of variation (CV) of VPD between sites and across different monitoring years (Spearman *r* = 0.86; *P* < 0.05, Fig. 5). This suggests that increasing variability in atmospheric aridity (VPD) increases seasonal photosynthesis-growth decoupling. Similar results were found with evaluating seasonal photosynthesis-growth coupling against the interaction of precipitation and temperature variability (fig. S17). We use the CV of VPD and air temperature and precipitation interaction as a metric of climate homogeneity during the course of the year relevant to photosynthesis-growth coupling across space and time (i.e., sites and years) as raw values of environmental variables are strongly site dependent (fig. S8). Growth and GPP are not fully decoupled during seasonal phase I as some GPP does occur during this phase. Since the greatest GPP-growth decoupling occurs during seasonal phase III where little to no growth happens during this phase, we also evaluated the relationship between the CV of VPD and CV of air temperature-precipitation interaction against the fractional length of seasonal phase III only. We found strong positive relationships between the phase III and the CV of VPD (Spearman *r* = 0.79, *P* < 0.05) and phase III and the CV of the interaction between air temperature and precipitation (Spearman *r* = 0.75, *P* = 0.06) (fig. S18).

## DISCUSSION

The term growing season is often used interchangeably with the period of carbon uptake in vegetation (*20*, *21*). This assumption, often characterized using remote sensing, eddy covariance, or meteorology, implies that photosynthesis and growth are always coupled. We found that while aboveground, woody biomass growth started synchronously with canopy expansion and photosynthesis (*31*, *63*), growth activity culminated by the end of July (Fig. 3 and figs. S4, S7, and S11). This was considerably earlier than photosynthetic activity, which typically concluded in late October through early November (Fig. 3 and figs. S4, S7, S9, and S10). Considering a more rigorous definition of the growing season to be the annual period when plants produce new tissue regardless of net carbon gain (*20*), the photosynthetic season and aboveground woody biomass growing season are decoupled for the studied North American temperate deciduous oak species. We therefore recommend clearly defining the two terms and refraining from using them interchangeably without careful consideration particularly in forested environments.

Because of their susceptibility of large earlywood vessels to embolism (*64*), often during winter freeze-thaw cycles (*65*), oaks predominantly rely on the relatively large xylem vessels formed in current year earlywood for the water transport needed for foliar expansion and stomatal conductance required for photosynthesis (*66*). This is clearly illustrated by anatomical microcore images showing the progressive development of earlywood vessels early in the growing season (fig. S11) and by relative conductive area trajectories that reflect a rapid early-season increase in conductive area followed by stabilization once vessel formation ceases (fig. S12). To protect their new xylem from embolism induced by xylem water tension during freeze-thaw cycles, leaf emergence in oaks often commences later than other species (*66*). Despite the later start to the active season, oaks generally achieve peak growth rates and terminate annual growth earlier than co-occurring diffuse porous and conifer species (*67*–*69*). As a result of this, xylem development and annual growth in oaks is more sensitive to spring and early summer climatic conditions and less so to late summer to autumn (i.e., post-July) climate (Fig. 2 and figs. S2 and S3) (*67*–*73*).

Our results also indicate that growth and GPP were temporally offset from each other not just at the seasonal scale but also at diel scales. Growth activity occurred primarily during phase A at the diel scale, between late nights and early mornings (00:00 and 07:00 hours) when temperature, VPD, and TWD, a form of reversible stem shrinkage linked to water deficit, were relatively low (Fig. 4 and fig. S13). By contrast, GPP peaked between late morning and solar noon, in concert with increasing light availability, at higher VPD and temperature during phase B at the diel scale (*74*). At the same time, increasing TWD during phase B suppressed growth activity. These results related to nighttime growth linked to turgor limitation are consistent with findings from automated dendrometry studies conducted since the early 20th century (*74*–*78*) through to more recently published literature (*43*, *79*). As a result of this temporal decoupling at daily timescales, when aggregated to seasonal timescales, growth activity occurs at lower temperatures and VPD than GPP (Fig. 3, E and F, and fig. S6). In addition, tree MDS_norm_ was strongly correlated with both daily maximum VPD and temperature (figs. S15 and S16). This provides further supporting evidence that these variables may regulate growth activity by influencing tree water status (potential) and turgor in cambial cells (*80*–*82*). At one site, Tonzi Ranch–CA, we also found that growth and GPP occurred under similar SWC niches (fig. S14). This suggests that although soil moisture may be key driver of the GPP and growth phenology and the magnitude of productivity (*62*), it may not be a mechanistic driver of the GPP-growth decoupling observed here. However, these comparisons were made only at one site during one active season, and, therefore, there remains a need in future work to more thoroughly investigate the potential role of SWC (and soil water potential) in influencing photosynthesis-growth decoupling.

Similar seasonal offsets between growth and GPP have been observed for temperate deciduous oaks in Europe (*41*) and for multiple Northern Hemisphere conifer species (*83*–*85*). Greater environmental sensitivity and faster down-regulation of sink compared to source activity under arid conditions has also been described at other sites (*35*, *36*, *43*, *67*). In nonwoody plants, this has also been inferred on the basis of a continued build-up of NSCs in the form of hexose and sucrose sugars under drought conditions (*40*), although this needs future research in woody plants (*85*). Moreover, because of larger vessel sizes, ring-porous oaks have to withstand greater xylem tensions than diffuse-porous hardwood species, which implies that developing cambium cells in oaks are also more likely to be limited by diurnal losses in hydraulic conductance and turgor pressure (*86*). Last, across sites and years, the degree of temporal photosynthesis-growth decoupling was strongly related to the annual dispersion in atmospheric aridity (VPD) and temperature-precipitation interaction, with higher aridity dispersion leading to increased decoupling (Fig. 5 and fig. S17). This suggests that larger intraannual variability in VPD and temperature-precipitation interaction leads to a shorter synchronous period favorable for both photosynthesis and growth to co-occur plausibly because of less conducive conditions for growth compared to photosynthesis. As VPD and drought extremes become more intense and frequent under climate change (*47*, *49*), such increased variability may negatively affect tree growth and forest carbon sequestration. At the same time, we note that our offset decoupling metric is based on the duration in days of coupled growth and GPP relative to the entire active season. This was because the sun-induced fluorescence (SIF)–derived GPP dataset that we used ended in 2021 and not all sites had flux towers. Consequently, we were only able to calculate the actual amount of growth (in seasonal phase I) and GPP (in seasonal phase III) for four (*4*) of the seven (*7*) site years (fig. S5). In future work, we recommend also emphasizing and investigating the environmental drivers of the actual amount of growth before full canopy expansion (seasonal phase I) and GPP assimilated subsequent to growth cessation (seasonal phase III).

Our results help contribute to explaining a puzzling conundrum of how and why deciduous oaks continue to prioritize photosynthetic activity during the summer by leaving stomata open (i.e., are anisohydric) despite increasing water stress and higher xylem vulnerability to embolism via cavitation (*48*, *64*, *87*, *88*). Here and in previous works (*89*, *90*), we find that once leaves are fully expanded, oaks maintain the capacity for photosynthesis through the entire active season into autumn (text S1 and figs. S9 and S10). However, they complete a majority of their annual growth by spring and early summer (by early July), and little to no growth occurs in late summer and autumn (late-July onward). It is likely that warm temperatures, high VPD, and depleted soil moisture particularly during the late summer (figs. S8 and S14) decreases turgor and inhibits aboveground growth (cell division and expansion) independent of current carbon assimilation (*10*, *42*, *54*, *71*, *91*). Consequently, during the period of down-regulated growth but sustained photosynthesis at the seasonal scale (phase III in Fig. 3 and figs. S4 and S7), it is possible that oaks prioritize continued assimilation and building up of NSC reserves and the allocation of carbon to other sinks (such as belowground growth, reproduction, and respiration) despite the increased risk of damage to their xylem as current-year vessels will likely embolize or occlude anyway before the next season (*64*, *66*, *70*). This is consistent with the hypothesized role of stomatal regulation of canopy conductance toward whole plant carbon coordination mediated through NSC dynamics while balancing the availability for environmental resources such as water (*42*, *80*, *92*, *93*). At the same time, we acknowledge that here, we primarily consider the biophysical responses of GPP-growth (i.e., source-sink) coupling to temperature, precipitation, and VPD. However, annual GPP and growth productivity and the start and end of the photosynthetic and growing seasons themselves are controlled by a complex interplay of environmental (e.g., temperature, photoperiod, and moisture), physiological (e.g., NSC accumulation, and hormonal), and genetic (e.g., ontogenic) factors, and these variables may also in turn influence source-sink coupling (*81*, *82*, *85*, *92*, *94*).

Many temperate and boreal conifer species have been shown to build-up NSC reserves in the latter period of the active season after the cessation of growth activity and use these assimilates to commence primary growth and secondary growth of earlywood in the following year (*83*, *95*). On the basis of radiocarbon analysis in both conifers and angiosperms (specifically oak), it has been shown that up to 50% earlywood carbon could have been assimilated from 1 to 2 years previously but that sources older than that are unlikely to be used for woody biomass growth (*96*, *97*). A study at two temperate deciduous sites in eastern North America that included oak species studied here found strong correlations between current year growth and integrated carbon assimilation over the current and previous year based on eddy covariance (*37*). In support of these findings, we note that earlywood growth, particularly for *Quercus rubra*, showed a significant (*P* < 0.01) positive relationship with prior year July through September SPEI (fig. S3). This suggests that humid and cool environmental conditions in late summer through autumn may facilitate higher photosynthetic activity and a build-up of NSCs promoting increased earlywood growth through the utilization of these reserves in the subsequent year (*31*, *93*). These results also highlight the potential for intraannually resolved tree-ring data (e.g., earlywood, latewood, quantitative wood anatomy, isotopes) to provide further insights into environmental and physiological memory effects on growth needed to more comprehensively resolve source-sink relationships and discrepancies. Nevertheless, these specialized tree-ring measurements currently represent a small fraction (less than 5%) of publicly available tree-ring data and the overwhelming majority of these specialized collections (~90% or more) remain biased toward gymnosperm species (*98*–*100*).

In our study, we investigate the temporal coupling between photosynthesis and aboveground woody biomass growth across intra to interannual timescales at which they remain understudied. We focus on multiple temperate deciduous angiosperm oak species across North America. Angiosperm species store most global plant carbon but remain underrepresented in xylogenetic and dendrochronological studies due to their more complicated wood structure when compared to gymnosperm species (*11*, *29*, *99*, *101*). We recognize that when considering woody biomass growth, there is a need in future work to consider cell wall thickening and lignification that may continue to occur after cambial cell division, and expansion is complete as dendrometers are unable to resolve these processes (*69*, *102*–*104*). At the same time, from a carbon perspective, lignification of woody biomass is likely less important in angiosperm species than for gymnosperm species. This is because the lignin fraction and fraction of woody biomass carbon stored as lignin (cf. cellulose and hemicellulose) is lower in angiosperm species (lignin mass fraction: ~18 to 25% and lignin carbon fraction: ~30 to 35%) than in gymnosperm species (lignin mass fraction: ~25 to 35% and lignin carbon fraction: ~40 to 45%) (*105*). Further, cell enlargement, cell wall formation, and lignification processes remain poorly studied for angiosperms species (*106*, *107*), and there is some evidence that they may be more strongly coupled in angiosperms (*106*, *108*–*111*). Microcores collected at Lamont Sanctuary–NY in September 2021 indicated fully mature and lignified latewood xylem cells suggesting a coincident end of season for cell enlargement, cell wall thickening, and lignification for this species at this study location (fig. S19). That being said, the lags between cell enlargement, cell wall thickening, and lignification and the extent to which they co-occur (or not) and how these lags may differ across species, years, and sometimes even within the same growth ring remain poorly constrained in angiosperm species (*102*, *106*, *107*, *112*). Further, we note that respiration, foliage, NSC storage and dynamics, belowground growth, root exudation, mycorrhizal associations, and herbivory are all major carbon-intensive processes or sinks that we do not resolve in this study (*29*, *69*, *93*, *95*, *103*, *104*, *113*, *114*). Installing a diversity of sensors such as sap flow sensors and dendrometers on more trees within the footprint of a flux tower together with in situ hyperspectral remote sensing, NSC measurements, xylogensis monitoring via quantitative wood anatomy, and process-based modeling will allow us to better evaluate how photosynthesis-growth relationships vary across species, age classes, dominance status, and site-level differences in microclimate, topography, and nutrient availability. They will also help us better evaluate memory effects related to prior environmental conditions and legacy effects of environmental stressors (e.g., heat waves and drought) on source-sink coupling, which may have the potential to either exacerbate or ameliorate ecosystem responses to stress (*9*, *30*, *35*, *37*, *41*, *53*, *83*, *84*, *92*, *115*, *116*).

In conclusion, we show that carbon assimilation and aboveground woody biomass growth are systematically decoupled in oaks across diel through seasonal timescales. Growth occurs during narrower environmental windows and is more sensitive to atmospheric aridity and temperature than photosynthesis at our study sites. Increases in aridity that inhibit both photosynthesis and growth have been implicated as a continued challenge facing oaks across North America and in forests globally (*48*, *117*, *118*). As climate change increases, the frequency and intensity of high-VPD events, heat waves, and droughts (*47*, *48*), our results highlight that the capacity of forests to sequester carbon over decadal to centennial scales may depend not just on carbon assimilation via photosynthesis but also their ability to allocate carbon toward woody biomass growth. If carbon allocation to long-lived woody pools declines more rapidly than assimilation, such a divergence may shift carbon flow toward more transient pools that have shorter residence times and diminish the long-term carbon sink capacity of forests (*22*, *44*, *71*). ESMs that assume consistently tight coupling between photosynthesis and growth may therefore overestimate future forest carbon sequestration under rising atmospheric moisture demand.

## MATERIALS AND METHODS

A conceptual figure of the experimental design is provided in Fig. 1 and fig. S1 and is described here in further detail.

### Growth

#### G1. Point dendrometers

Point dendrometers sensors were mounted on tree sapwood to provide micrometer-level measurements of aboveground radial growth and hydration status (*54*). Raw dendrometer data were quality checked and adjusted for artefacts such as outliers and large nonbiological jumps or drops [e.g., (*119*)]. Following initial data processing, raw tree radius data in micrometers were converted to growth and TWD using the “zero growth” concept (*59*). A growth occurrence was recorded for a tree each time its dendrometer trace superseded all previous maxima (*59*). Subsequently, TWD was calculated as the difference (in micrometers) between a growth value and the current recorded value until a new growth occurrent was recorded (*59*). TWD_norm_ was calculated as the ratio between TWD of a tree and its 95th percentile TWD value (*80*). MDS_norm_ was calculated as the difference between the maximum and minimum dendrometer value each day (i.e., MDS), standardized by the 95th percentile MDS value (*80*). A total of 19 (Morton Arboretum–IL), 8 (Lamont Sanctuary–NY), 3 (Pace Forest–VA), and 5 (Tonzi Ranch–CA) mature trees were outfitted with one automated point dendrometer per individual. As far as possible, dendrometers were installed at breast height (~1.3 m) on the north side of the tree or at a location without direct incident sunlight. Dead bark was scraped off to mitigate the effects of bark and sensor expansion, and dendrometers were installed as close to the living cambium as possible. Dendrometers at Lamont Sanctuary–NY, Pace Forest–VA, and Tonzi Ranch–CA were manufactured by Hise Scientific and by TOMST at Morton Arboretum–IL. At Pace Forest–VA and Tonzi Ranch–CA, outfitted trees were located proximal to and within the footprint of the eddy covariance flux tower.

#### G2. Xylogenesis monitoring and wood anatomical analysis

Five healthy *Quercus* spp. trees were selected within the Lamont Sanctuary–NY, to monitor the dynamics of xylem formation during the 2021 active season. These included two *Quercus alba* and three *Q. rubra* individuals. These trees were the same individuals previously equipped with automatic dendrometers as part of ongoing monitoring activities at the site. Microcores (1.8 mm in diameter) containing bark, cambium, and developing xylem were extracted every 15 days between March and September 2021 using the Trephor tool (*120*). To minimize tree damage, the sampling followed a spiral checkerboard pattern around the stem, maintaining a minimum distance of 4 to 6 cm between consecutive sampling points (*121*). The biweekly sampling was a trade-off between having sufficient temporal resolution to distinguish cellular scale division and growth progression in xylem anatomy, labor intensive laboratory work, and avoiding excessive tree damage by partial girdling. Immediately after collection, the microcores were stored in FAA solution (formalin–acetic acid–alcohol, 5:5:90 by volume) for 1 week to ensure adequate fixation of tissues in angiosperms, and subsequently transferred to 70% ethanol for long-term storage.

In the laboratory, samples were gradually dehydrated in an ethanol series (50, 70, 85, and 95%) and embedded in JB4 acrylic resin (Polysciences, Germany), following the manufacturer’s protocol. Embedded blocks were trimmed to expose the sample and eliminate excess resin and then sectioned transversely to a thickness of 10 μm using a WSL custom-built sliding microtome (WSL, Switzerland). Histological sections were stained with a double-staining solution consisting of 1% safranin and 0.5% Astra Blue, allowing clear differentiation between lignified and nonlignified tissues. After staining, the sections were rinsed with distilled water and passed through increasing ethanol concentrations (50 and 95%). Last, sections were permanently mounted on glass slides using Eukitt mounting medium (Sigma-Aldrich, USA).

Digital images of the stained cross sections were captured using a Nikon optical microscope equipped with an LU Plan Fluor 10×/0.30 objective and connected to a high-resolution digital camera operated through the NIS-Elements imaging software (Nikon Instruments Inc.). Quantitative anatomical analyses were carried out using ROXAS software (v3.0.31) (*122*, *123*), focusing primarily on lumen area (La) as a proxy of hydraulic functionality and vessel formation over the growing season.

#### G3. Tree rings

Tree ring data were downloaded from the International Tree Ring Data Bank on 1 April 2024 or collected and processed by coauthors using standard dendrochronological methods (*124*). See table S1 for more information. Sites were broadly dispersed across the distributional range of each species (Fig. 2) compiled by (*125*) and available from (*126*). Criteria for inclusion of tree ring data were that they belong to one of the eight study species (Fig. 1) and that the earliest and latest years must at least span the 1950 to 2000 CE period. The eight study species were selected on the basis of tree species present at the high-resolution monitoring sites for which dendrometer data were collected. To preserve high-frequency (i.e., interannual) variability related to climate and eliminate lower frequency (e.g., decadal) variability and biological growth trends, tree ring data were detrended using a Friedman Smoother and converted to standardized RW indices (*127*, *128*). Following this, we evaluated the relationship between the 1-month SPEI (*129*) and annual tree growth. To do so, we calculated the correlation between monthly 1-month SPEI and annual growth between 1950 and the last year of growth for each month starting with prior year January to current year December. We chose 1-month SPEI for these calculations since we were most interested in the shortest possible climate window at the monthly scale to which tree growth is most responsive. These correlations are therefore quite “conservative,” as correlations of annual tree growth and monthly climate generally increase when climate data are averaged across seasonal timescales (*130*). The Pearson correlation for each month was calculated as the median of 1000 bootstrapped Pearson correlations. Draws were made with replacement while preserving the order of sampling of the climate and growth data. These tree growth response functions help evaluate the monthly climate windows to which tree growth is and is not sensitive.

### Canopy phenology and GPP

#### P1. Phenological data from in situ remote sensing

Phenological cameras or PhenoCams were installed at all four sites (*131*). These cameras capture images at periodic intervals (approximately every 15 min) in the red (R), green (G), and blue (B) bands allowing us to calculate a metric called the GCC as G/(R + G + B) that is closely related to canopy phenology and plant photosynthetic capacity (*131*, *132*). For each image, we calculated GCC values over a defined region of interest that included the forest canopy and trees instrumented with dendrometers. We excluded all images with solar angles of less than 10°. PhenoCams at Lamont Sanctuary–NY and Tonzi Ranch–CA were manufactured by StarDot NetCam SC and set up and analyzed using protocols outlined by (*131*). At the Pace site, GCC was calculated using hyperspectral reflectance measured by the Fluospec2 spectrometer system pointing at a *Q. alba* with dendrometer installed (*133*).

#### P2. Eddy covariance

Measurements were collected using flux towers at two sites (Tonzi Ranch–CA and Pace Forest–VA) and integrated to 30-min intervals. These data were then gap filled and quality controlled to exclude values that were outside ranges or conditions with insufficient air turbulence. Eddy covariance directly monitors Net Ecosystem Exchange (NEE) above an ecosystem. We partitioned NEE into its two constituent components, GPP and ecosystem respiration (ER), by extrapolating night-time NEE values using their relationship with temperature as a proxy for daytime ER (*134*, *135*) using the OneFlux pipeline (*136*). Tonzi Ranch–CA has two colocated flux towers for the overstory and understory. The understory tower primarily samples grassland vegetation while the overstory tower samples overall ecosystem productivity from both grasses and trees. To isolate and estimate the fraction of annual GPP for the forest at Tonzi Ranch–CA, we calculated the difference between integrated daily GPP estimates of the tall overstory and short understory tower (*7*).

#### P3. Satellite remote sensing

SIF signals emitted by chlorophyll-a of vegetation can be observed by satellites and are closely coupled to plant photosynthetic performance and have been found to be well correlated with ecosystem scale GPP (*58*, *137*–*140*). Here were derived daily GPP estimates in 2021 at the ecosystem scale for two study sites, Morton Arboretum–IL and Lamont Sanctuary–NY, that did not have in situ flux towers. We use the dataset generated by (*58*, *141*) that harmonized and downscaled satellite measurements of SIF obtained by the TROPOMI spectrometer onboard the Copernicus Sentinel-5P mission with half-hourly flux tower GPP across North American ecosystems to a 500-m spatial resolution. The overpass time is 13:30 local solar time. The comparatively early start of the photosynthetic season and larger variability in the annual cumulative GPP cycle in California in Fig. 2 is because these sites are predominantly woody savannah ecosystems. Graminoids typically green-up and commence photosynthetic activity earlier in the year than oak trees and may contribute a substantial fraction of ecosystem scale annual GPP (*89*). Further, mixed contributions from both vegetation types and lower SIF signals leads to lower signal-to-noise ratios and higher satellite retrieval uncertainties in woody oak-savannahs (*58*, *141*). This limitation of satellite remote sensing in woody savannahs is addressed using the dual-tower approach to eddy covariance at Tonzi Ranch–CA described above. A limitation worth mentioning particularly in relation to the phenology of GPP at the Eastern US tree-ring sites is that while most sites are oak dominated, signals retrieved by the SIF sensor likely include contributions from other species (e.g., *Acer* spp., *Betula* spp., *Fagus* spp., *Pinus* spp., etc.) present on the landscape.

#### P4. Pulse amplitude modulated fluorometry

We used the Junior PAM portable chlorophyll fluorometer (Heinz Walz GmbH) to evaluate leaf-level photosynthetic performance at Lamont Sanctuary–NY. Top-of-canopy sun-facing leaves were collected from three oak trees also outfitted with dendrometers using a pole-saw on average every 3 days (59 leaves per tree, 177 total) between April and November 2021 (figs. S9 and S10). Leaves were typically collected around 8:45 a.m. local time, kept moist, and dark adapted for at least 2 hours. Subsequently, rapid light curves (RLCs) were performed on each leaf. An RLC measures the leaf-level chlorophyll fluorescence response to nine different increasing actinic radiance levels from 0 through 845 μmol photons·m^−2^·s^−1^. See (*142*) for a more detailed explanation of the RLC protocol and text S1 for descriptions of the studied fluorescence parameters. These data were used to evaluate whether early season increases in GPP during spring and late season decreases in GPP during autumn were primarily a function of physiological changes at the leaf level, changing photosynthetic surface area, or changing environmental conditions (text S1 and figs. S9 and S10).

#### Dispersion in climate

Dispersion in climate parameters, including temperature, precipitation, and VPD was calculated as the annual CV of each variable, after averaging them to the monthly scale. Temperature and VPD data were collected in situ at all sites at 15-min intervals. We first calculated daily averages for both temperature and VPD followed by monthly averages. Daily precipitation data at all four sites were obtained from ERA-5 reanalysis (*143*) as Morton Arboretum–IL and Lamont Sanctuary–NY did not have in situ rain gauges. Last, annual dispersion was calculated as the CV of average monthly temperature, precipitation, and VPD for each year.
