# Supplementary material for: Decoupled carbon assimilation and growth responses to aridity in temperate deciduous oaks
Source: Sci Adv. 2026 Jun 12;12(24):eady7139. doi: 10.1126/sciadv.ady7139 (PMC13262636; doi:10.1126/sciadv.ady7139)
Supplement: Supplementary file 1 — Supplementary Text Figs. S1 to S19 Legend for table S1 [file sciadv.ady7139_sm.pdf]

Science Advances
AAAS

Supplementary Materials for

Decoupled carbon assimilation and growth responses to aridity in temperate deciduous oaks

Mukund Palat Rao *et al.*

Corresponding author: Mukund Palat Rao, mukund@ldeo.columbia.edu

Sci. Adv. **12**, eady7139 (2026)
DOI: 10.1126/sciadv.ady7139

The PDF file includes:

Supplementary Text
Figs. S1 to S19
Legend for table S1

Other Supplementary Material for this manuscript includes the following:

Table S1

Supplementary Text

1. Rapid Light Curves and leaf-level chlorophyll fluorescence

At one site, Lamont Sanctuary-NY, we ran chlorophyll fluorescence Rapid Light Curves (RLCs) on dark-adapted top-of-canopy sun-exposed leaves to compare leaf-level photosynthetic capacity and performance with stand scale greenness estimated from a co-located PhenoCam and satellite remote sensing derived ecosystem-scale metrics of carbon assimilation (Supplementary Figure 9-10). We were particularly interested in using these data to investigate two questions. The first was to understand whether the increase in GPP during seasonal Phase I in spring of canopy-emergence between April and May was principally a function of increasing photosynthetic surface area as leaves expanded along with more favourable environmental conditions for photosynthesis or if physiological changes at the leaf-level may also be contributing to increases in GPP. The second, was to investigate whether the declines in GPP after August observed at the ecosystem-scale during seasonal Phase III were related primarily to less favourable environmental conditions for photosynthesis or to physiological changes at the leaf-level (e.g., declining chlorophyll concentrations, progressive seasonal degradation of leaf photosynthetic machinery) leading to decreased foliar photosynthetic capacities and lower ecosystem-scale GPP.

A total of 177 RLCs were performed on three mature oak individuals also outfitted with dendrometers beginning with initial bud-burst and leaf emergence in April through to leaf senescence and abscission in November 2021 (59 RLCs/tree in total performed on average every three days). We found that all photosynthetic performance parameters (photosynthetic efficiency or the maximum photochemical yield of photosystem II: F_v/F_m, non-photochemical quenching: NPQ, maximum electron transport rate: ETR_{max}, quantum efficiency: α, yield of photosystem II: Φ_{PSII}) increased between April and May synchronously with leaf emergence and expansion (Supplementary Figure 9-10). After this initial increase foliar photosynthetic efficiency (F_v/F_m), known as the maximum photochemical yield of photosystem II (PSII), which represents the proportion of absorbed light energy that can be used for photosynthetic electron transport (photochemistry) in a dark-adapted state (*121*), remained highly stable at around ~0.8 or higher from May through October indicating a maintenance of high photosynthetic capacity during this period. Other photochemistry parameters (ETR, ETR_{max}, α, Φ_{PSII}) continued to increase from June through September and peaked in September. The period of leaf senescence between late October and early November then coincided with large declines in photosynthetic efficiency (F_v/F_m) and other photochemistry parameters (ETR, ETR_{max}, α, Φ_{PSII}). Lastly, NPQ which is related to excess absorbed light energy dissipated as heat (i.e., not used for photochemistry to drive photosynthesis) and is related to the degree of photoprotection in leaves (*121*), was highest in May coincident with full foliar expansion (highest PhenoCam GCC and NDVI), and then gradually decreased through November. Taken together, these results suggest that the increase in GPP during the period of bud-burst and leaf expansion (i.e., primary growth) between April and May (seasonal Phase I) is not solely a function of increasing foliar surface area and more favourable environmental conditions for photosynthesis but is likely also related to physiological changes in the leaf as they develop their photosynthetic apparatus. On the other hand, since trees maintained high photosynthetic capacities at leaf-level (i.e., high F_v/F_m, ETR_{max}, and α) until late October when leaves began to change colour and senesce, declines in GPP after August during seasonal Phase III are more likely to be related to less favourable environmental conditions than to leaf physiological changes. This might particularly be relevant to the decline in GPP observed in September since ETR and Φ_{PSII} were the highest and NPQ was relatively low across all study trees indicating the capacity for high

photosynthetic performance and a low degree of photoprotection during this period. Site 1 (Morton Arboretum-IL) and Site 3 (Pace Forest-VA) are mesic temperate hardwood oak dominated ecosystems similar to Site 2 (Lamont-Sanctuary-NY) where RLC measurements were made. We believe that similar physiological mechanisms might exist at these two sites, but the lack of available data preclude us from making more definitive statements. On the other hand, Site 4 (Tonzi Ranch-CA) is a semi-arid location where precipitation is unevenly distributed during the course of the year and peaking in the winter and spring months between November and March (Supplementary Figure 8) (*60*). Previous studies at Site 4 (Tonzi Ranch-CA) have shown that oaks trees at this site also maintain their capacity to assimilate carbon through the summer and the reduction in GPP during the summer is linked to environmental drivers leading to a decrease carboxylation capacity of Rubisco and not to leaf damage (*60*, *79*).

2. Wood anatomical data

To complement high-resolution dendrometer monitoring and to validate that seasonal trends in above-ground woody biomass radial stem expansion corresponded with cellular-scale xylem development, we collected biweekly microcores from five mature *Quercus* spp. trees instrumented with point dendrometers at the Lamont Sanctuary-NY from March through September 2021 (see Methods). The wood anatomical analysis focused on the quantification of the Relative Conductive Area (RCTA), as a proxy for the formation of early and latewood vessels and overall hydraulic functionality.

Microcores collected in early March showed no visible signs of cambial reactivation or cell enlargement, indicating dormancy (Supplementary Figure 11). A steep increase in RCTA occurred between early April to mid-May, Day of Year (DOY) 90 and 130, reflecting the onset of cell division and rapid expansion of large earlywood vessels essential for canopy development (Supplementary Figure 12). This early peak in conductive area is consistent with the need to reestablish hydraulic continuity and support leaf-out, particularly in ring-porous species such as oaks where prior year vessels are often non-functional due to embolism or occlusion (*64*, *66*).

The maximum RCTA values were observed in early May (between DOY 130–150), after which a gradual decline was recorded despite continued presence of developing vessels. This decline reflects the transition from earlywood to latewood formation, where vessel size decreases and total conductive area plateaus or diminishes. While our staining protocol (1% Safranin + 0.5% Astra Blue) allowed visualization of lignified and partially lignified tissues, we did not attempt to explicitly classify the timing of lignification, given the overlapping nature of enlargement and secondary wall formation in angiosperm species (*90*, *91*) (Supplementary Figure 19).

The smoothed seasonal trajectory of RCTA (Supplementary Figure 12) mirrors the phenological phases described in the main text at the seasonal scale (Figure 3 and Table 1). This includes a rapid early-season increase during seasonal Phase I (coinciding with canopy expansion), stabilization during Phase II, and limited changes thereafter during Phase III when radial growth ceases but photosynthesis continues. Importantly, our anatomical data support the hypothesis that the majority of hydraulic capacity is constructed early in the growing season. This is consistent with a strategy in ring-porous oaks to front-load investment in stem conductivity early in the season, possibly as a response to decoupling between source (photosynthesis) and sink (growth) processes later in the year due to increasing aridity (*29*). Individual tree data (Supplementary Fig. 11-12)

confirm these trends, with slight variation among species (e.g., *Q. rubra*, *Q. alba*) but consistent early peaks in RCTA followed by stabilization or decline. Smoothing with cubic splines or generalized additive models yielded comparable patterns. These results emphasise that the physiological window for significant anatomical development of new vessels is temporally constrained to the first half of the active season, reinforcing our broader finding that carbon allocation to above-ground woody biomass growth terminates earlier than photosynthesis in temperate oaks.

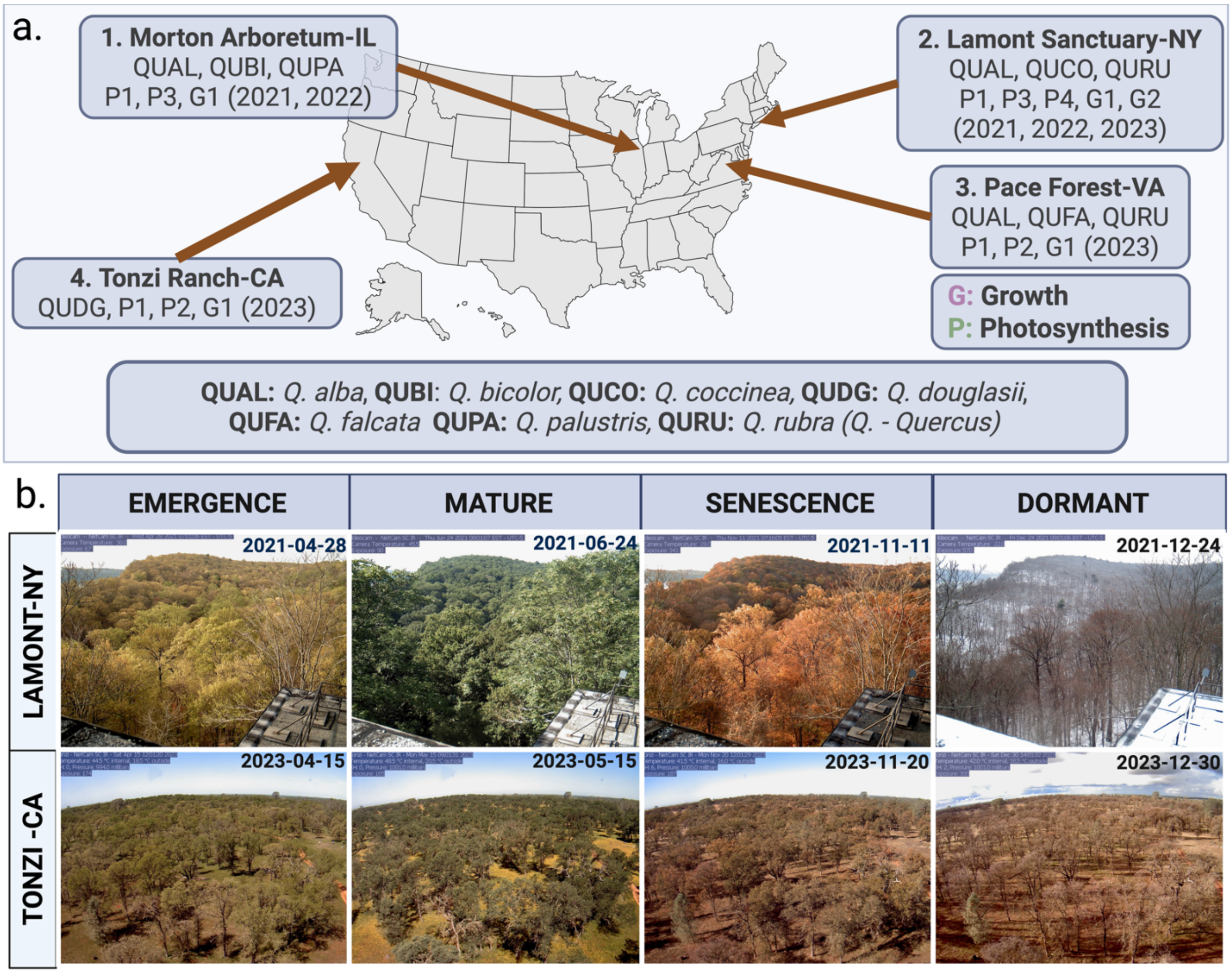

Supplementary Figure 1. Data streams used to evaluate photosynthesis-growth coupling at four high-resolution monitoring sites shown in **Main Text Figure 1**. **a.** Inset map shows the locations of monitoring sites along with study years, species, and measurements made at each site. Sites are 1. Morton Arboretum-IL (41.82°N, 88.07°W), 2. Lamont Sanctuary-NY (41°N, 73.90°W), 3. Pace Forest-VA (78°N, 33°W), and 4. Tonzi Ranch-CA (38.43°N, 120.96°W). Photosynthesis related data include PhenoCams (P1), eddy covariance (P2), satellite remote sensing (P3) and leaf-level chlorophyll fluorescence (P4). Growth data include point dendrometers (G1) at all sites and wood anatomy (G2) at Lamont-NY only. **b.** Four PhenoCam images that are broadly representative of seasonal canopy phenological stages at Lamont-NY (a mesic temperate deciduous hardwood forest similar to Morton-IL and Pace-VA) and Tonzi-CA (a semi-arid Mediterranean oak-savannah). Created at BioRender.com. IL: Illinois, NY: New York, VA: Virginia, CA: California.

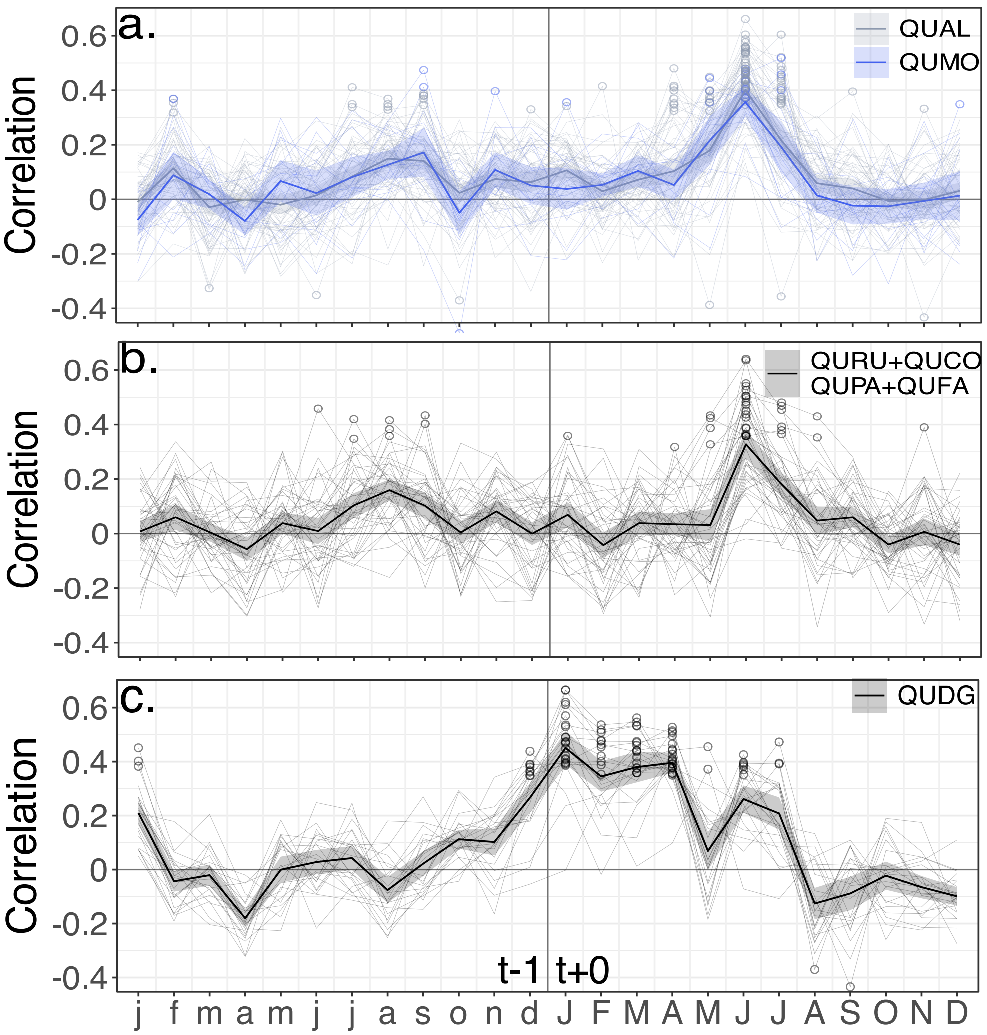

Supplementary Figure 2. Climate sensitivity of tree radial growth across a network of 137 oak (*Quercus spp.*) total annual tree ring width (RW) series in North America. a. Pearson correlation between RW in the current year and 1-month SPEI starting from prior year (lag t-1) January through current year (lag t+0) December for QUAL (n=59 series, in grey) and QUMO (n=15 series, in blue). The solid line is the median correlation across all sites and the shaded interval represents ±2 standard errors. Open circles represent significant correlations (p<0.01, 2-sited t-test) for a particular series and 1-month SPEI for that month. Correlations are calculated between 1950 and the last year of growth for each series while 1-month SPEI is calculated as the mean for a 0.5x0.5 grid box around the coordinates of each site. Months are abbreviated to the first alphabet with lower case letters for the prior year. **b.** The same as in **a.,** but averaged for QURU, QUCO, QUPA, and QPFA RW series (n=40 series). **c.** The same as in **a.** and **b.,** but for QUDG (n=23 series). See **Main Text Figure 2** and **Supplementary Table 1** for locations of sites. The fraction of sites with a significant (p<0.01) correlation between growth and 1-month SPEI are shown in **Main Text Figure 2 (b-d-f)**. SPEI: Standardised Precipitation Evapotranspiration Index, QUAL: *Quercus alba*, QUMO: *Quercus montana*, QURU: *Quercus rubra*, QUCO: *Quercus coccinea*, QUPA: *Quercus palustris*, QUFA: *Quercus falcata*, QUDG: *Quercus douglasii.*

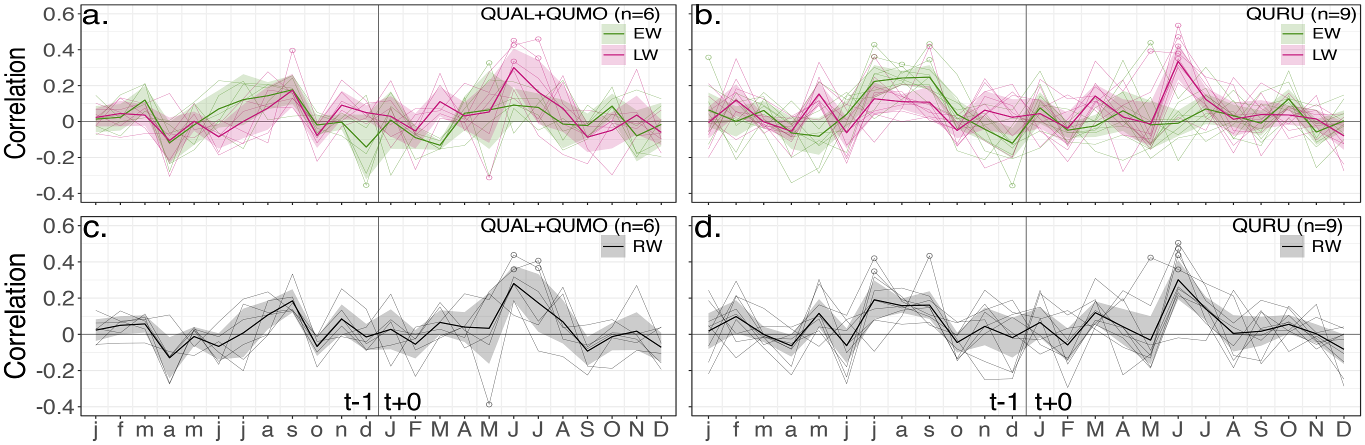

Supplementary Figure 3. Climate sensitivity of tree radial growth for a subset of series with earlywood (EW), latewood (LW), and total annual ring width (RW) measurements. a. Correlation between annual EW and LW measurements (in green and purple) in the current year and 1-month SPEI starting from prior year (lag t-1) January through current year (lag t+0) December for QUAL and QUMO (n = 6). The solid line is the median correlation across all sites and the shaded interval represents ±2 standard error. Open circles represent significant correlations ($p<0.01$, 2-sited t-test) for a particular series and 1-month SPEI for that month. Correlations are calculated between 1950 and the last year of growth for each series while 1-month SPEI is calculated as the mean for a 0.5x0.5 grid box around the coordinates of each site. Months are abbreviated to the first alphabet with lower case letters for the prior year. **b.** The same as in **a.,** but averaged for QURU series (n = 9). **c.** and **d,** as in **a.** and **b.,** but for the RW measurements for the same series. Comparing a. and c together with b. and d., we observe that EW growth is mildly associated with prior year summer-autumn climate conditions (between July and September) particularly for QURU while LW is most sensitive to current year June conditions for QUAL+QUMO and QURU. RW climate sensitivity resembles LW sensitivity but with slightly weaker correlations.

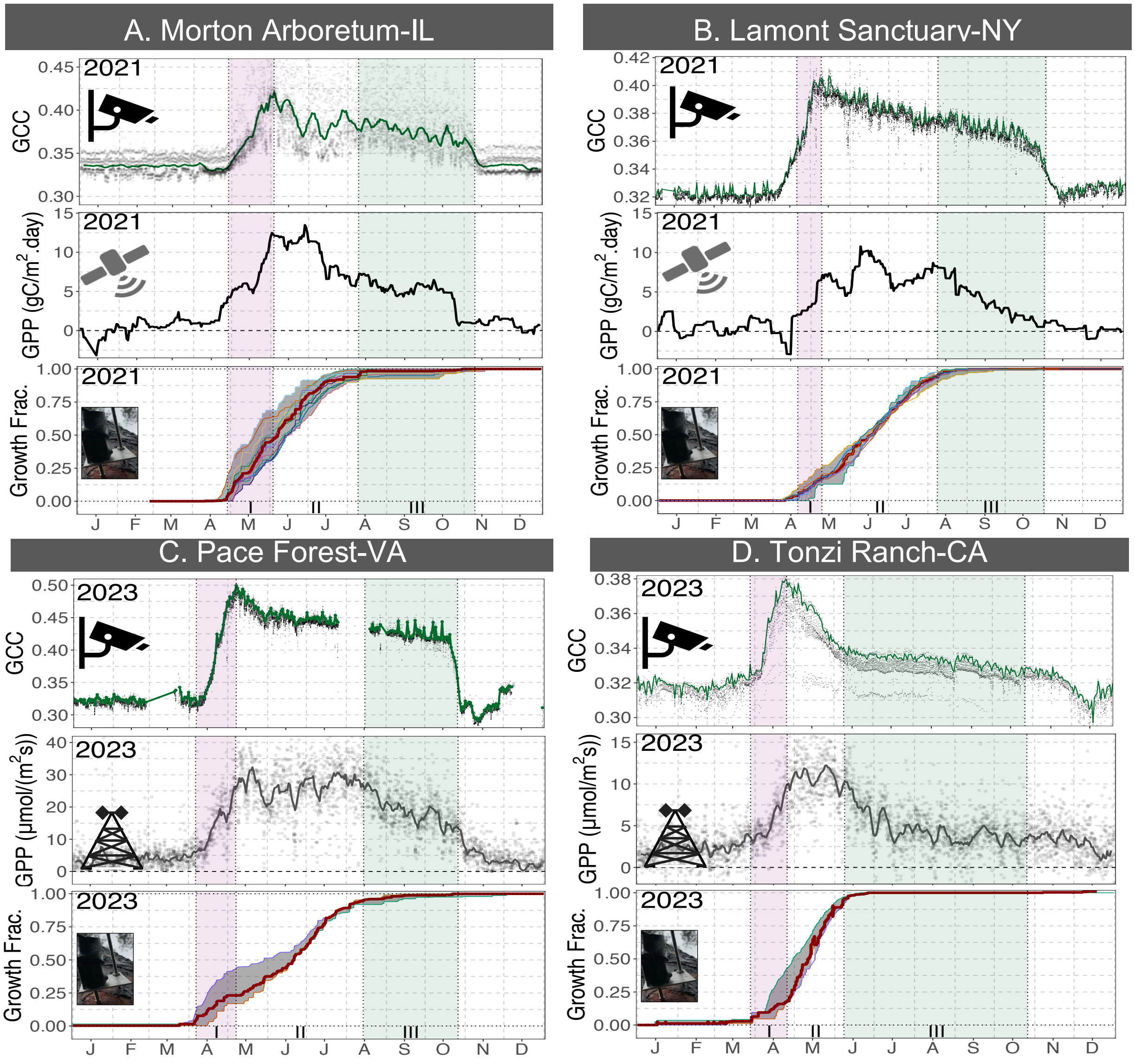

Supplementary Figure 4. Seasonal photosynthesis-growth decoupling at four oak forest sites in North America based on differing phenologies of PhenoCam derived canopy Green Chromatic Coordinate (GCC), gross primary productivity (GPP), and point-dendrometer derived fraction of annual above-ground radial growth. A. Morton Arboretum-IL in 2021, **B.** Lamont Sanctuary-NY in 2021, **C.** Pace Forest-VA in 2023, and **D.** Tonzi Ranch-CA in 2023**.** GPP estimates are derived from satellite remote sensing of solar-induced chlorophyll fluorescence (SIF) at the Morton Arboretum-IL (A) and Lamont Sanctuary-NY (B) and using eddy covariance estimates of half-hourly carbon-dioxide fluxes above the canopy at Pace Forest-VA (C) and Tonzi Ranch-CA (D). GCC: Dark green line is daily 90th percentile, GPP: in C and D the solid black line is daily mean between 10:00 and 14:00 hours with a 5-day smoothing, Growth: in dark red is the median across all monitored trees and undulations are a result of a short intervals of missing data for one or more dendrometer series (i.e., no gap-filling or interpolation). The active season is divided into three phases. **Phase I** (purple shaded area): leaf expansion (primary growth) together with above-ground radial growth (secondary growth) but GCC inferior to its annual maximum, **Phase II:** concurrent GPP and radial growth with maintenance of fully expanded foliage and canopy, and **Phase III** (green shaded area): continued canopy greenness and GPP but downregulated radial growth, followed by canopy senescence. X-axis labels represent abbreviated months of the year. Main Text Figure 3 combines information from each data stream (GPP, GCC, growth) onto one plot. GCC dots in Main Text Figure 3 are a 5-day running mean of the solid green curve here scaled using average value prior to leaf emergence (as 0) and the maximum value attained during active season (as 1). Smoothed GCC line in Main Text Figure 3 is a double logistic regression fit (smoothing spline at Tonzi Ranch-CA), GPP is the cumulative annual fraction of all daily GPP data, and median growth curve remains same across both figures.

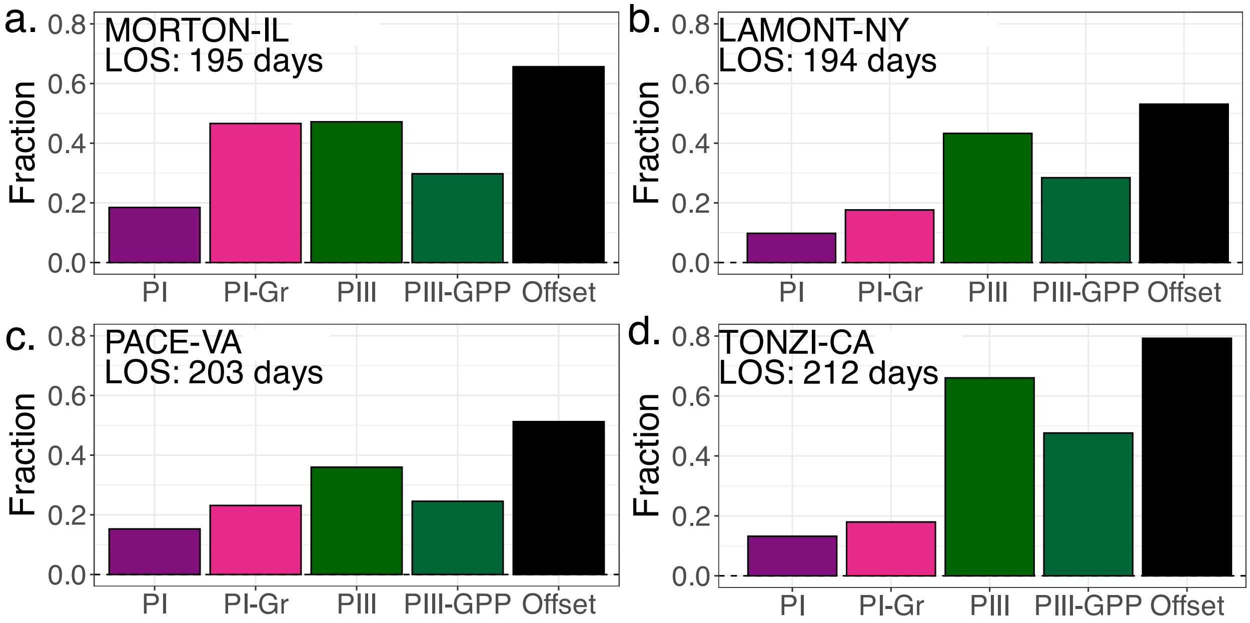

Supplementary Figure 5. Metrics regarding the degree of coupling between GPP and growth during the active season based on Main Text Figure 3 a-d. Data for Morton-IL and Lamont-NY are from 2021 and for Pace-VA and Tonzi-CA are from 2023. (LOS = total number of number of days in Phase I+II+III).

i. PI and PIII represent the fractional lengths of Phase I and Phase III respectively relative to the entire active season.

ii. PI-Gr represents the percent of annual growth that occurs during Phase I

iii. PIII-GPP the percent of annual GPP that occurs during Phase III.

iv. Offset is a metric of the seasonal decoupling between GPP and growth (calculated as PI+PIII) with a higher Offset representing a shorter Phase II relative to the total length of the active season

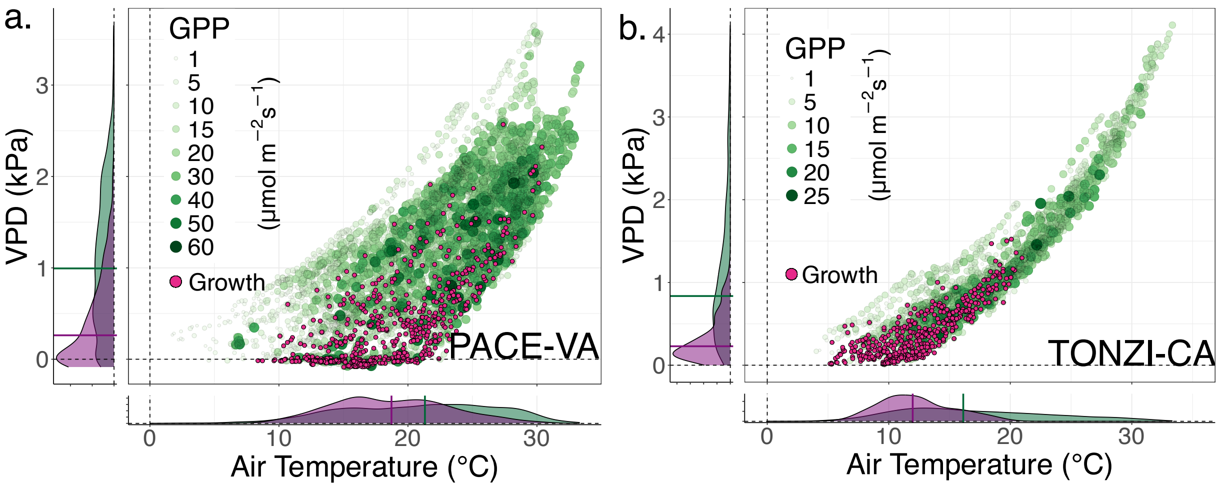

Supplementary Figure 6. Growth occurs over a narrower climatic niche of cooler temperature and lower Vapour Pressure Deficit (VPD) than Gross Primary Productivity (GPP) across seasonal scales. a. Environmental sensitivity of GPP (in green) and growth (in purple) as a function of air temperature and VPD at Pace Forest-VA. Circle sizes scale with GPP. Kernel density plots show temperature and VPD distributions for growth and GPP along with vertical bars for medians. Median temperature and VPD are 21.3°C and 0.99 kPa for GPP and 18.7°C and 0.26 kPa for growth. **b.** As in a. but at Tonzi Ranch-CA. Median temperature and VPD are 16.1°C and 0.83 kPa for GPP and 12°C and 0.23 kPa for growth. Comparisons are for all hours between Phase I and Phase II in Main Text Figure 3 when growth and GPP co-occur and excludes Phase III when GPP continues with little to no growth (Apr-06 to Aug-14 at or Pace Forest-VA and Mar-28 to June-08 for Tonzi Ranch-CA in 2023). Growth occurs when a dendrometer records a new maximum value that supersedes all prior maxima (Zero-Growth Concept, see Methods). Growth occurrences are shown for all dendrometer monitored trees at each site. Note that axis scales differ between left and right hand side panels.

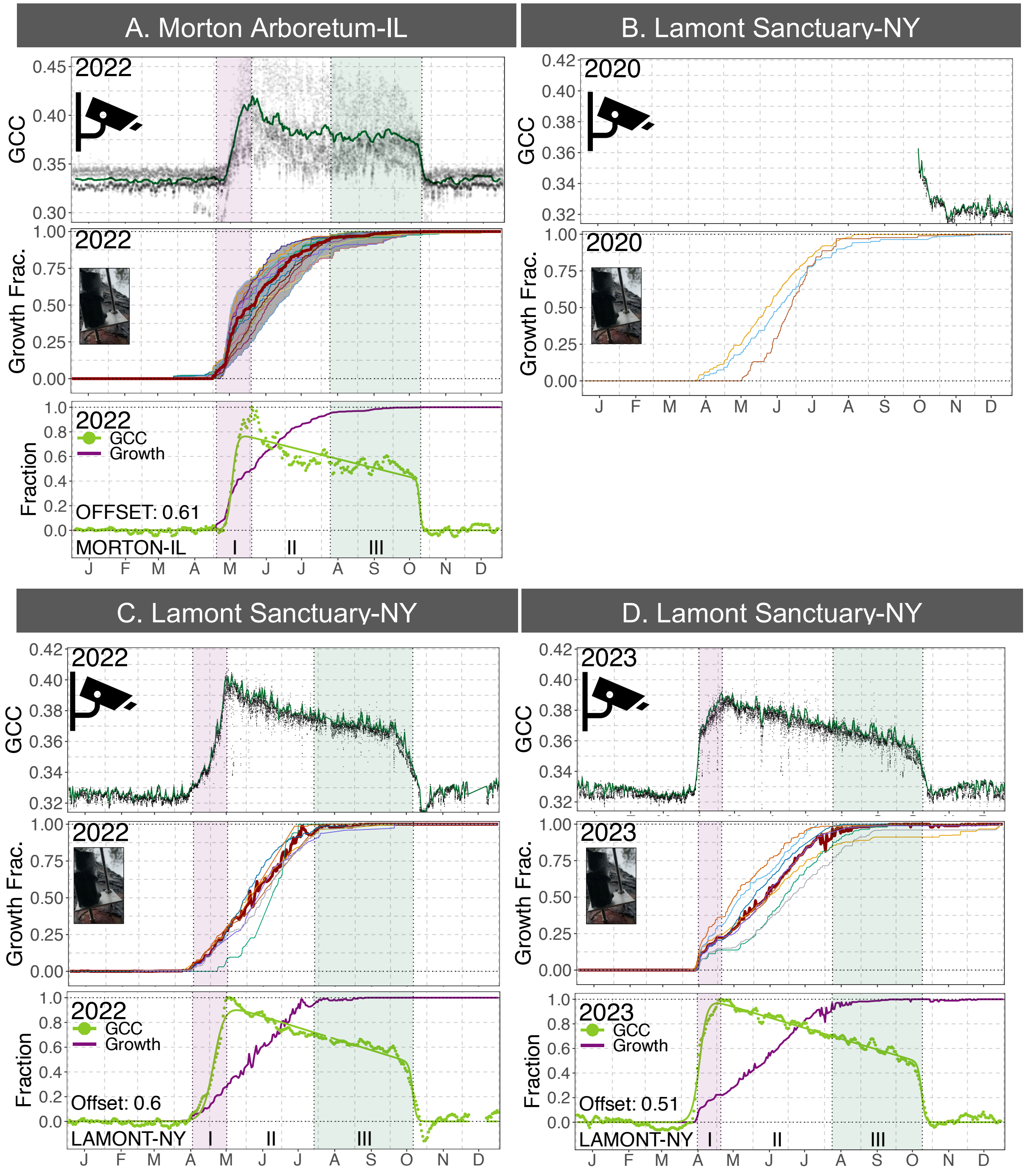

Supplementary Figure 7. Similar to Figure 3 but for phenology of GCC and fraction of annual above-ground radial growth at two oak dominated forest sites in North America for other monitoring years. A. Morton Arboretum-IL in 2022, **B.** Lamont Sanctuary-NY in 2020, **C.** Lamont Sanctuary-NY in 2022, and **D.** Lamont Sanctuary-NY in 2023**.** No GPP were available for these years since the Turner et al. GPP ends in 2021 (see Methods) and these sites do not have flux towers. This is also why we used the peak in annual GCC (and not GPP) do define the boundary between seasonal Phase I and Phase II. Lower panel in A, C and D combines GCC and growth. GCC dots shown in the combined GCC-Growth subplot are a running 5-day mean of solid green curve scaled between 0 and 1 to which a double-logistic regression was fit. The Lamont Sanctuary-NY PhenoCam was installed in 2020 and while these data are not used in analyses, they qualitatively show that the 'growing season' ended prior to the 'photosynthetic season' even in 2020 despite the partially incomplete dataset acquired that year.

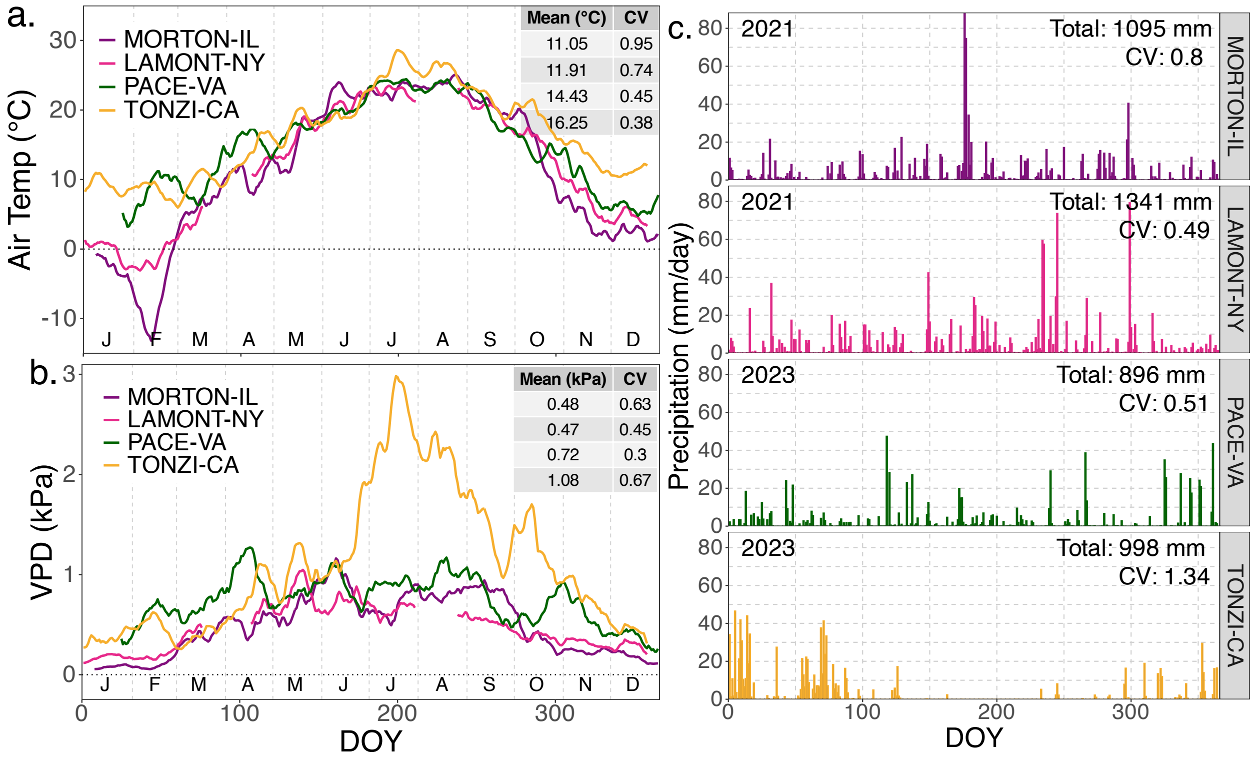

Supplementary Figure 8. Climatic data at the four high resolution monitoring sites. a. Air temperature (°C), **b.** vapour pressure deficit (VPD in kPa), and **c.** Precipitation (mm). Mean annual temperature, mean annual VPD, total annual precipitation and the intra-annual coefficient of variation (CV) of the three variables are also described. Temperature and VPD data are smoothed with 15-day running averages for better visualisation. Data presented are for 2021 at Morton Arboretum-IL and Lamont Sanctuary-NY and 2023 at Pace Forest-VA and Tonzi Vaira-CA. DOY: Day of Year. Months are abbreviated to the first alphabet. Note that precipitation data at the sites were obtained from ERA-5 reanalysis since Morton-IL and Lamont-NY did not have in-situ rain gauges. Further, as not all sites had soil moisture and groundwater data, we were unable to assess how they may influence photosynthesis-growth coupling except at Tonzi Ranch-CA where in-situ soil moisture data was available.

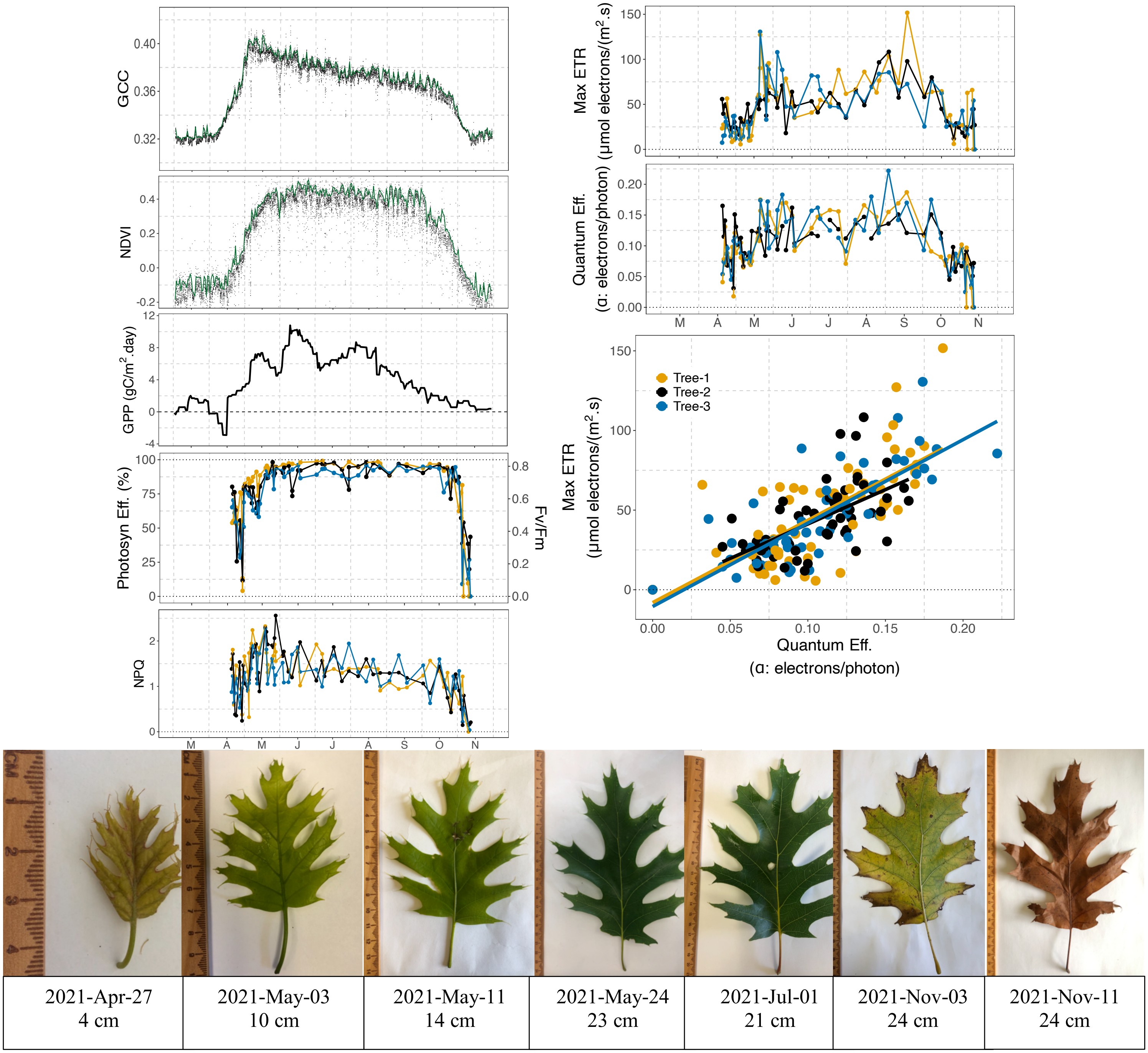

Supplementary Figure 9. Leaf photosynthetic capacities developed between spring foliar emergence and expansion and were maintained through senescence in autumn at Lamont Sanctuary-NY. Comparison between two PhenoCam derived indices (Green Chromatic Coordinate: GCC and Normalised Difference Vegetation Index: NDVI) at the canopy scale, Gross Primary Productivity (GPP) at ecosystem scale estimated using satellite remote sensing of solar-induced chlorophyll fluorescence, and leaf-scale chlorophyll fluorescence parameters monitored on three oak individuals between April and November 2021 (Tree-1: yellow, Tree-2: blue, and Tree-3: blue). GCC and GPP data are the same as in Supplementary Figure 4. Leaf-scale fluorescence parameters include the Maximum Photochemical Yield of Photosystem II (F_v/F_m), Non-Photochemical Quenching (NPQ), Maximum Electron Transport Rate (ETR), and Quantum Efficiency (α). F_v/F_m compares minimal chlorophyll-a fluorescence in the dark-adapted state (F_o) with maximal chlorophyll-a fluorescence (F_m) after a saturating light pulse (10,000 μmol-photons/m^2s for 0.6s) with variable fluorescence F_v calculated as F_m-F_o. In general, F_v/F_m is between 0.79 and 0.84 for healthy non-

stressed leaves indicating that 79-84% of absorbed light energy is directed towards photochemistry. We also rescale F_v/F_m to photosynthetic efficiency such that an F_v/F_m of 0.84 is 100%. NPQ represents excess absorbed light energy dissipated as heat. Maximum ETR describes value at which the Rapid Light Curve saturates, quantum efficiency (α) describes the initial slope of the increase in ETR with increasing light levels (see Supplementary Figure 10). Photosynthetic parameters (F_v/F_m, NPQ, Maximum ETR, and α) increase synchronously with leaf emergence and expansion in the spring and remain stable though late-summer (September) before decreasing with autumn leaf senescence (October-November). Monthly averages are presented in Supplementary Figure 10. The scatter plot between quantum efficiency (α) and maximum ETR shows that the rate of the initial rise in the ETR-Photosynthetic Photon Flux Density (PPFD) curve is strongly coupled with the maximum ETR attained at the leaf-level as is expected for sun-exposed leaves. PPFD is the intensity of photosynthetically active radiation (PAR) in the ~400–700 nm spectral range that plants can use for photosynthesis. Also shown are few sample images of leaves which RLCs were performed (7 out of 177 total leaves measured) along with their collection dates and leaf lengths from the petiole to leaf tip. Leaves were fully expanded by late May after which maximal leaf length remained stable.

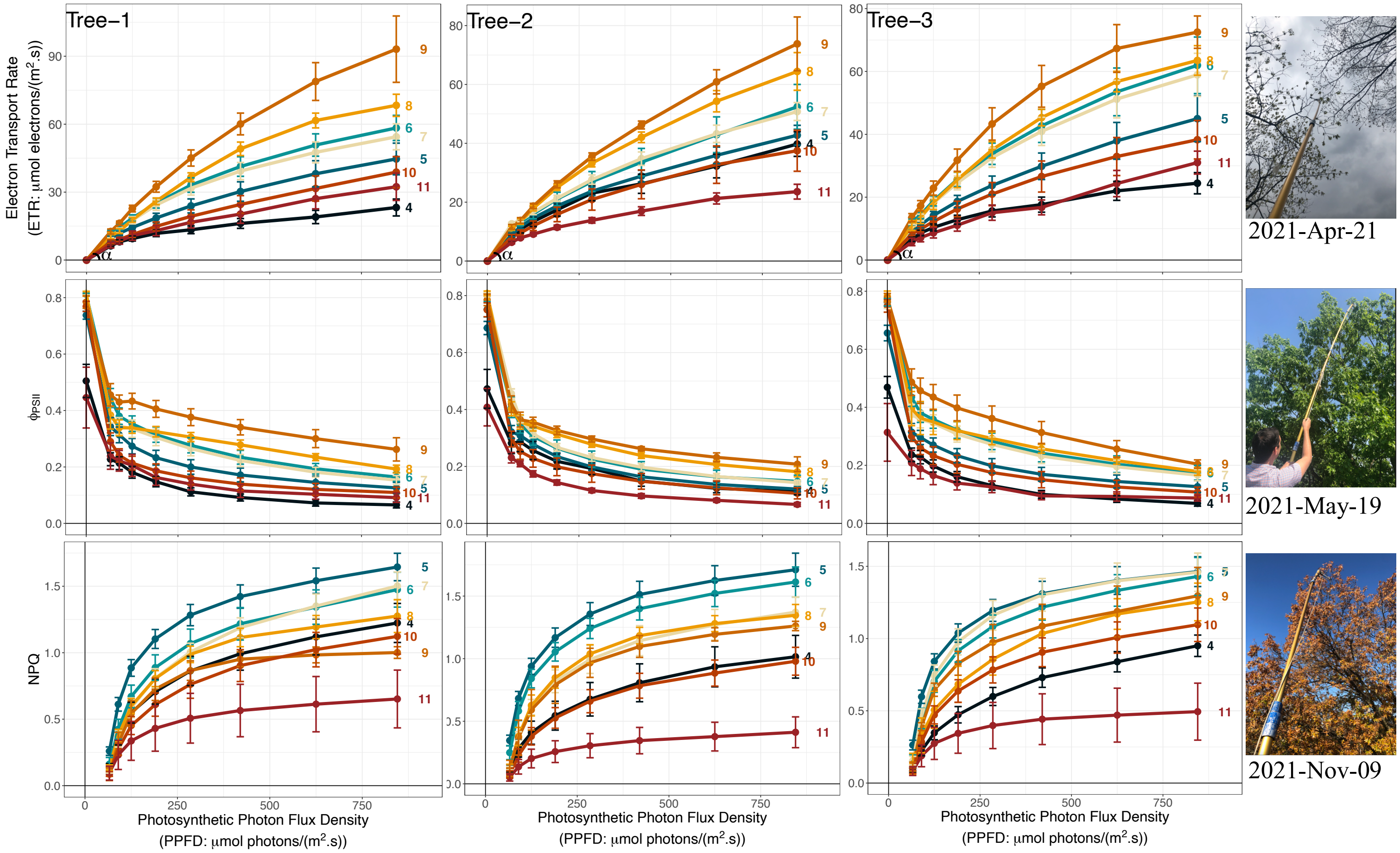

Supplementary Figure 10. Rapid Light Curves (RLCs) describing monthly means of Electron Transport Rate (ETR), Yield of photosystem II: Φ_{PSII}, and Non-Photochemical Quenching (NPQ) as a function of light intensity or Photosynthetic Photon Flux Density (PPFD). 59 RLCs/tree in total performed three oak trees (Tree-1, Tree-2, Tree-3) at Lamont Sanctuary-NY are described in the 3 columns. RLCs were performed on average every three days starting with leaf emergence in April through leaf senescence in November 2021. RLCs were subsequently averaged to the monthly scale to better visualise changes in plant photosynthetic performance capacity. Months average RLCs are described by numeric value (4–April, 5–May, 6–June, 7–July, 8–August, 9–September, 10–October, 11-November). ETRs and Φ_{PSII}, which are related the capacity for photosynthetic performance at the leaf scale increase between April (4) and September (9), peak in September, and then decrease in the autumn between October (10) and November (11). On the other hand, NPQ which is linked to leaf investment in photoprotection is highest immediately following leaf expansion in May (5) and decreases though the remainder of the active season. Measurements were made on dark-adapted top-of-canopy leaves. Some images of collections using a pole-saw at different leaf stages are shown on the right (leaf emergence: April, fully expanded leaves: May, leaf senescence: November).

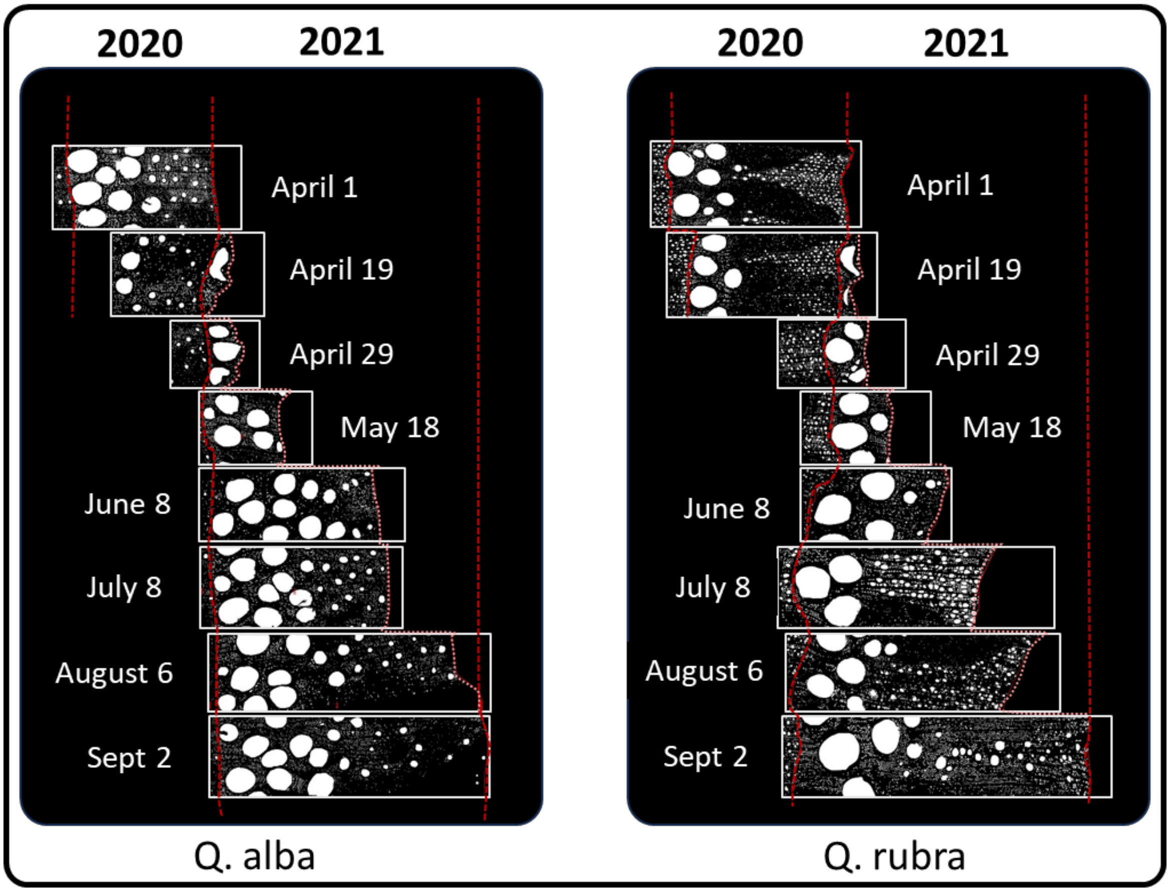

Supplementary Figure 11. Sequential images of xylem vessel development in two oak species based on anatomical microcores collected at Lamont Sanctuary-NY in 2021. Images show ROXAS-processed stem sections of *Quercus alba* (left) and *Quercus rubra* (right) collected during the 2021 growing season to monitor xylem development. Microcores were taken approximately every two weeks using a Trephor device. Although only 8 sampling dates are shown here, the full campaign extended before and after the growing season to ensure accurate detection of both onset and offset of xylem growth. The top row illustrates the fully developed xylem from the previous year (2020) as a reference. Earlywood growth and the formation of the large xylem vessels occurs between April and May coincident with leaf expansion (Supplementary Figure 4). Trees then switch to producing latewood between June and late August. Note that these images are qualitative as cores were collected in a checkerboard pattern across the tree and absolute ring sizes may differ. See Supplementary Figure 12 for quantitative data of conductive area over time. Red solid lines mark annual ring boundaries, and thin pink dotted lines indicate the progression of developing xylem across dates.

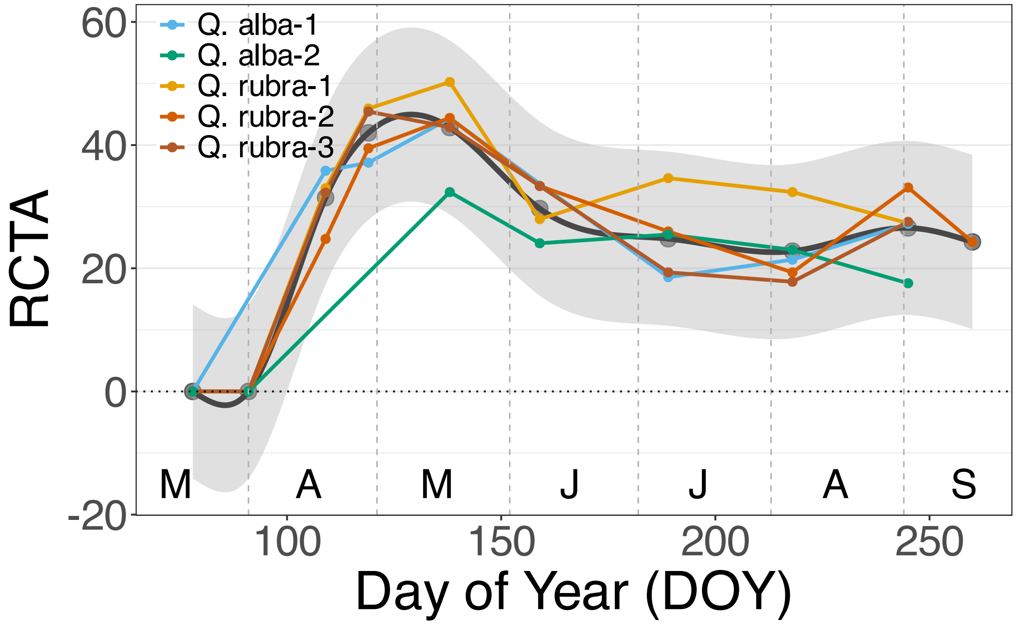

Supplementary Figure 12. Relative Conductive Area (RCTA) for five trees at Lamont Sanctuary-NY over the course of the formation of the 2021 annual growth ring. RCTA measures the total area of conductive vessels as fraction of total ring area formed over the same period. Earlywood is rich in large conductive vessels and RCTA is highest during the period of earlywood formation (April to mid-May). The latewood vessels in comparison are smaller and contribute less conductive area as a fraction of the growth ring (June to September). Different line colours represent different trees while the solid black line represents the Generalized Additive Model (GAM) smooth fit of RCTA as a function of DOY with a 95% confidence interval shown in grey.

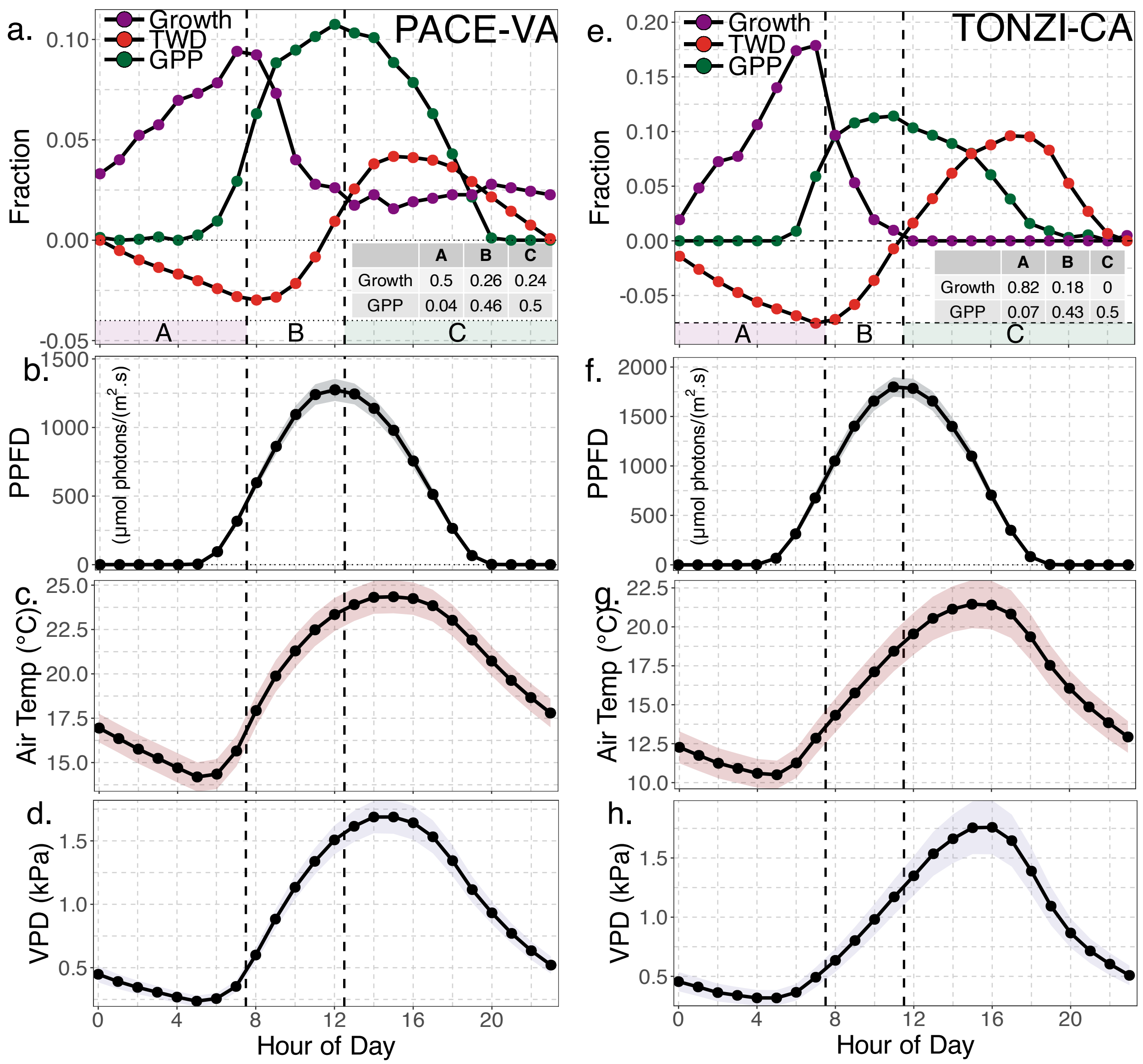

Supplementary Figure 13. Photosynthesis-growth decoupling at the diel scale. Growth occurs between late night and early morning (00 to 07 hours) over a narrower climatic niche of cooler temperature and lower Vapour Pressure Deficit (VPD) than Gross Primary Productivity (GPP) which peaks at or just prior to solar noon. a. Fraction of growth occurrences (green dots), fraction of GPP, scaled Tree Water Deficit (TWD), **b.** Photosynthetic Photon Flux Density (PPFD, µmol photons/m^{2}s), **c.** Air Temperature (°C), **d.** Vapour Pressure Deficit (VPD, kPD) across hours of the day (00 to 23 hours) during Seasonal Phase I and II (Apr-26 to Aug-14) at Pace Forest-VA in 2023. Shading in subplots b, c, and d ±2 standard errors of the mean. Subplots **e-h** follow the same order but are for Seasonal Phase I and II (Mar-28 to June-08) at Tonzi Ranch in 2023. Growth occurrence fraction is calculated across hours when a growth occurrence is registered on all monitored trees following the Zero Growth Concept (Zweifel et al. 2016). GPP and Growth fractions sum to one. Mean standardised TWD curve is derived by scaling daily TWD by the daily maximum TWD in comparison to midnight TWD. Standardised TWD is therefore related to reversible stem radial change and negative and positive standardised TWD values indicate and expanding and contracting stem respectively relative to TWD at midnight.We divide photosynthesis-growth coupling at the diel scale into three phases (Table 1). The table in subplots **a.** and **e.** describe the fraction of seasonal growth and GPP during Diel Phases A, B, and C. Note different y-axis scales between left and right panels.

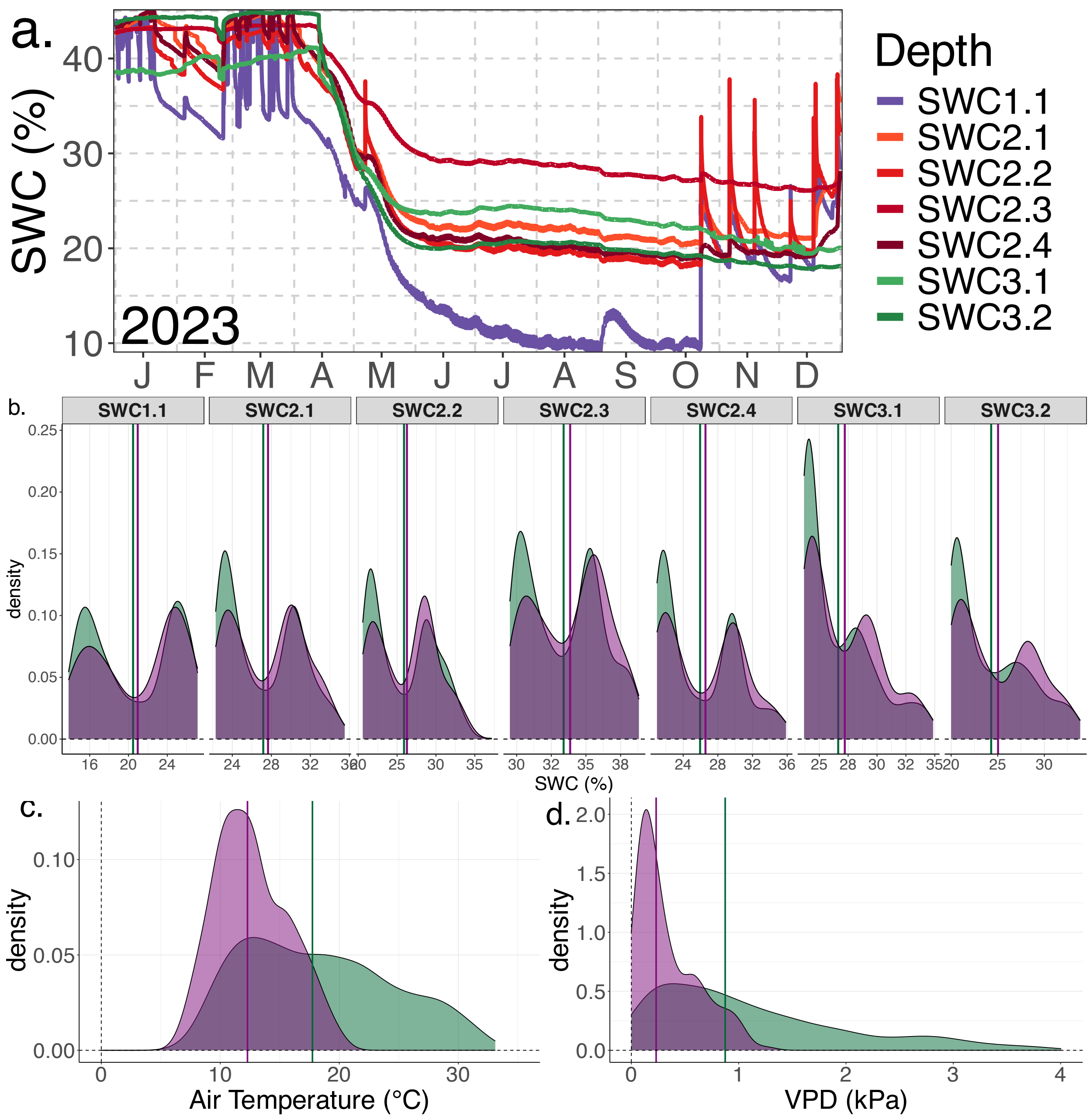

Supplementary Figure 14. Evaluating the potential role of Soil Water Content (SWC) in controlling photosynthesis-growth decoupling in comparison to air temperature and vapour pressure deficit (VPD) at Tonzi Ranch-CA. a. SWC percent on a volumetric basis (m^3/m^3) for 3 different depths with replicate measurements (SWC1.1 at -5 cm, SWC2.1 through SWC2.4 at -20 cm, and SWC3.1-3.2 at -55 cm). SWC is highest during the late winter through spring months (January-March) and decreases to an annual minimum by October. SWC is lowest closer to the surface (-5 cm). **b.** Kernel density distribution plots for growth (in purple) and gross primary productivity (GPP – in green) as a function of SWC distributions. Vertical bars indicate median SWC for growth and GPP at each measurement depth and replicate. Differences in median SWC for photosynthesis and growth are not significant based on 1000-bootstrapped samples of median with replacement for any depth or replicate although growth occurs at slightly higher median SWC (i.e., more moist soils) across all measurements. SWC2.1, SWC2.2, SWC2.3, and SWC2.4 are replicate measurements at -20 cm and SWC3.1 and SWC3.1 are replicate measurements at -55 cm. **c.** Growth and GPP distribution plots as a function of air temperature and **d.** VPD for comparison. The kernel density distribution in subplots **c.** and **d.** are the same as those integrated in Figure 3f of the Main Text. Comparisons in b, c, and d. are for all hours during seasonal Phase II at Tonzi Ranch-CA in 2023.

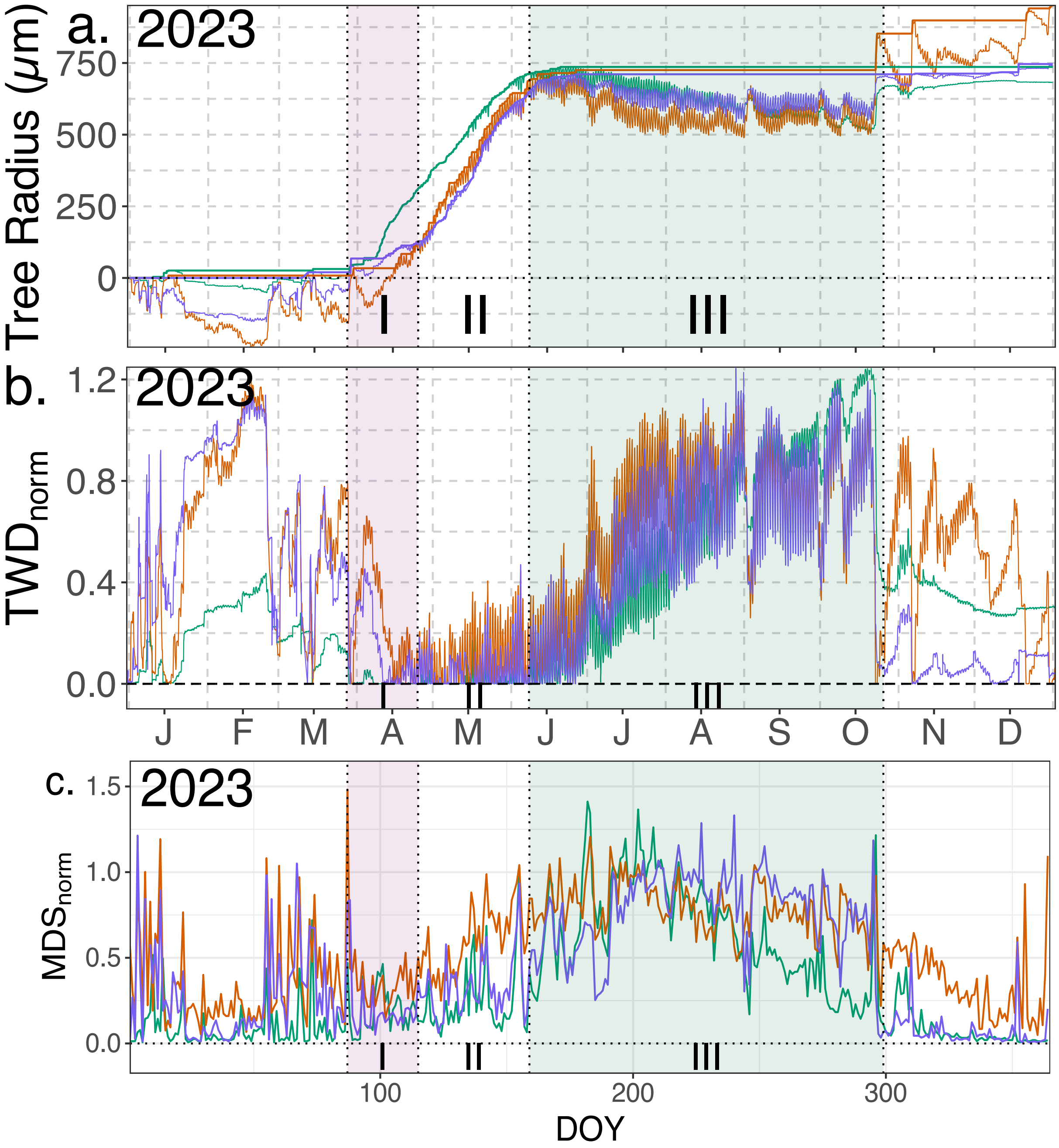

Supplementary Figure 15. Raw dendrometer traces and derived variables related to water status for three monitored blue oak (*Quercus douglasii*) trees at Tonzi Ranch-CA in 2023. a. Raw dendrometer traces of tree radius (µm) along with horizontal lines that indicate 'growth' based on the Zero Growth Concept (Zwiefel et al 2016). **b.** describes normalised Tree Water Deficit (TWD_{norm}), calculated as the difference between tree radius and the Zero Growth Concept (ZG) line in the top panel divided by the 95th percentile TWD value of each tree. **c.** Normalised Maximum Daily Shrinkage (MDS_{norm}), where MDS is calculated as the difference between the maximum and minimum dendrometer value each day, subsequently standardised by the overall 95th percentile MDS value. TWD_{norm} and MDS_{norm} allow us to more easily compare water stress across trees compared to absolute differences across trees in µm (Peters et al., 2025). Colours indicate different monitored trees.

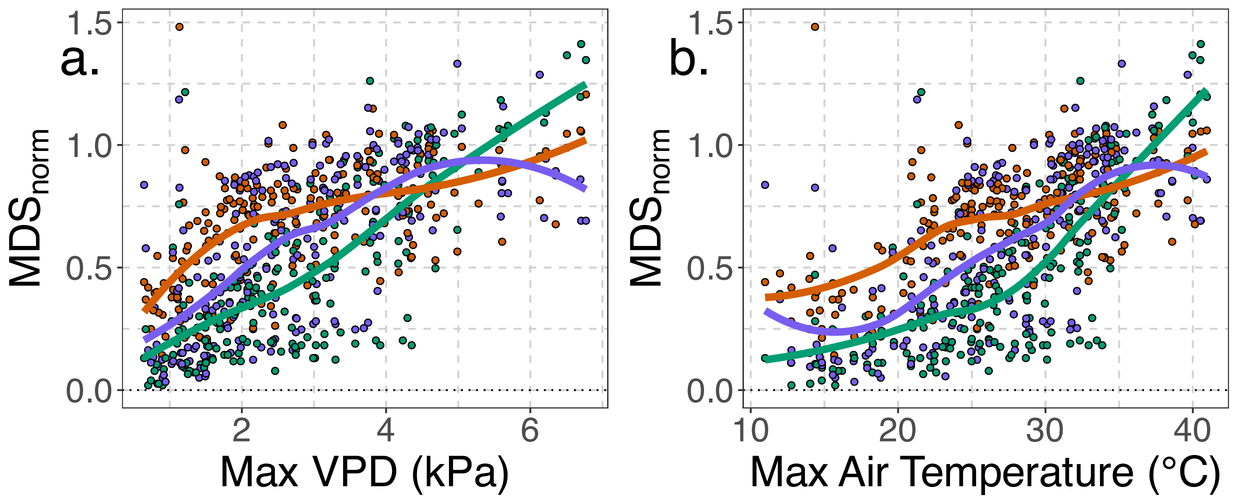

Supplementary Figure 16. Environmental drivers of Maximum Daily Shrinkage (MDS_{norm}). Scatter plot between MDS_{norm} and **a.** daily maximum vapour pressure deficit (VPD) and **b.** maximum air temperature. Three different colours (orange, purple, and green) indicate three different monitored trees. Best fit loess lines are also shown. Spearman Rank Correlations between MDS_{norm} and VPD for three trees are 0.74 (green), 0.57 (orange), and 0.69 (purple) and correlations between MDS_{norm} and maximum air temperature are 0.76 (green), 0.56 (orange), and 0.70 (purple). Relationships are similar with daily mean VPD and air temperature albeit marginally weaker. All correations are statistically significant ($p<0.01$, 2-sided t-test).

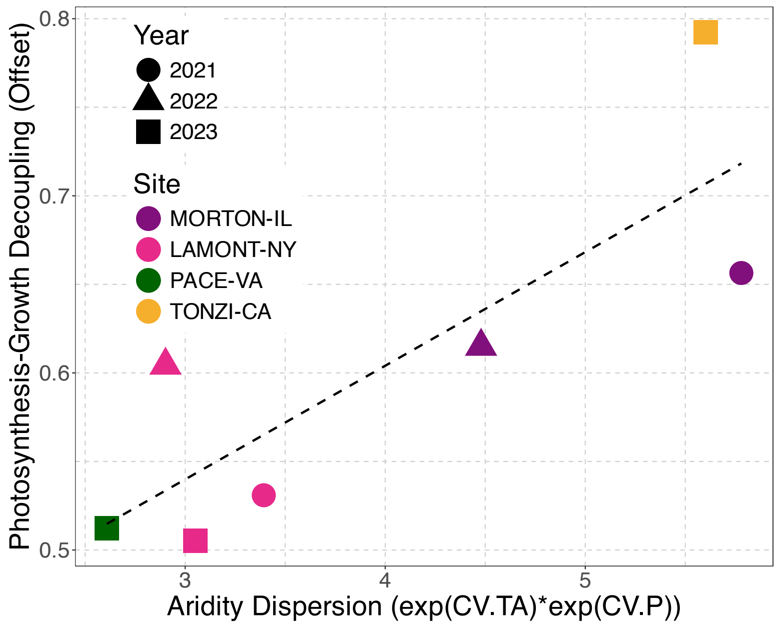

Supplementary Figure 17. Annual dispersion in aridity increases photosynthesis-growth decoupling. Relationship between the annual dispersion in aridity calculated as interaction between the coefficient of variation of air temperature (CV.TA) and precipitation (CV.P) and the fractional period of photosynthesis-growth offset (Offset). Offset represents the fraction of the active where growth and GPP are de-coupled or not fully co-occuring (fractional length of Seasonal Phase I and Phase III, relative to Phases I, II, and III). As annual aridity dispersion increases photosynthesis-growth decoupling increases (Spearman r=0.77, $p < 0.05$). Note that a fit of CV.TA+CV.P on log(Offset is equivalent to the fit of exp(CV.TA)*exp(CV.P) or exp(CV.TA+CV.P) on Offset. We prefer the latter for easier comparison with Main Text Figure 5. Note that in the legend, colours correspond to sites and shapes to site-years.

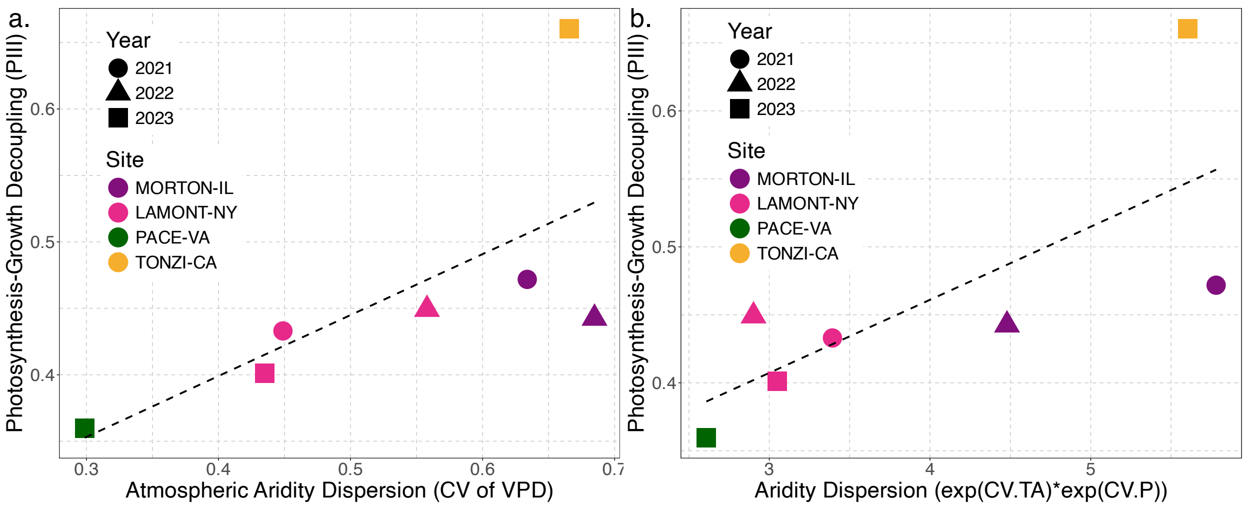

Supplementary Figure 18. Annual dispersion in aridity increases photosynthesis-growth decoupling. Relationship between the fractional length of Phase III (PIII) and annual dispersion aridity, measured by the annual coefficient of variation (CV) of **a.** mean monthly vapour pressure deficit (VPD) and **b.** air temperature (CV.TA) and precipitation (CV.P) interaction. PIII represents the fraction of the active where GPP continues but growth is complete (fractional length of Phase III, relative to Phases I, II, and III). As annual aridity dispersion) increases photosynthesis-growth decoupling increases (CV of VPD vs PIII Spearman r=0.79, $p<0.05$ and exp(CV.TA+CV.P) vs PIII Spearman r = .75, p = 0.06). This suggests that as aridity becomes more variable inter-annually the seasonal time period of the active season where photosynthesis/GPP continues to occur without growth increases (also see Main Text Figure 5 and Supplementary Figure 17). Note that in the legend, colours correspond to sites and shapes to site-years.

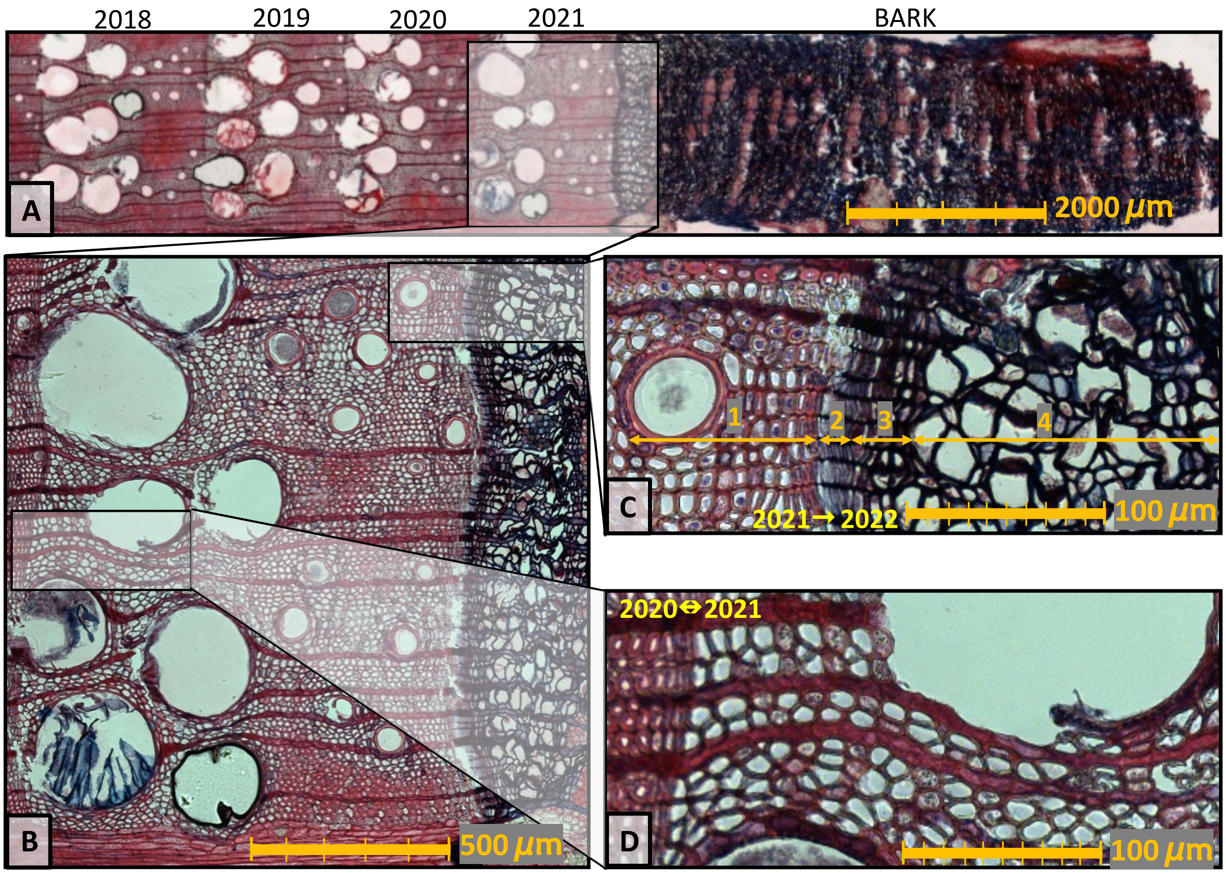

Supplementary Figure 19. Anatomical cross-section of a microcore from *Quercus rubra* (northern red oak) sampled on September 2nd (2021) illustrating the state of cambial activity and xylem differentiation at the end of the 2021 growing season. (**A**) Overview of the stem cross-section showing the bark and the last four annual growth rings. (**B**) Detailed view of the 2021 growth ring. (**C**) High-magnification image of the cambial region at the sampling date. Fully mature latewood xylem cells with thick secondary walls and narrow lumina are visible, showing intense safranin staining, indicating lignification. Arrows denote the main tissue zones: **(1)** xylem, **(2)** cambial zone, **(3)** functional phloem, and **(4)** collapsed phloem and onset of the bark. (**D**) Corresponding anatomical area from the previous year, shown for comparison, illustrating the similarity to the structural characteristics of the last-formed xylem cells at the end of the previous (2020) growing season. Our double-staining procedure that uses a solution of safranin and Astra Blue allows us to differentiate between lignified tissues which stain red and non-lignified tissues which stain blue. Close up images **B** and **C** suggest that by September (2021) cell division, and enlargement, cell wall thickening, and lignification were complete while the active phloem suggests continued transport of sugars from source organs (leaves) to storage organs (such as roots) driven by photosynthetic activity.

Supplementary Table 1. Metadata for *Quercus spp.* tree-ring data used including Site Names, Locations, Species, and the Contributor.
